# Integrated Toolset for WSN Application Planning, Development, Commissioning and Maintenance: The WSN-DPCM ARTEMIS-JU Project

**DOI:** 10.3390/s16060804

**Published:** 2016-06-02

**Authors:** Christos Antonopoulos, Katerina Asimogloy, Sarah Chiti, Luca D’Onofrio, Simone Gianfranceschi, Danping He, Antonio Iodice, Stavros Koubias, Christos Koulamas, Luciano Lavagno, Mihai T. Lazarescu, Gabriel Mujica, George Papadopoulos, Jorge Portilla, Luis Redondo, Daniele Riccio, Teresa Riesgo, Daniel Rodriguez, Giuseppe Ruello, Vasilis Samoladas, Tsenka Stoyanova, Gerasimos Touliatos, Angela Valvo, Georgia Vlahoy

**Affiliations:** 1 Applied Electronics Laboratory, Department of Electrical & Computer Engineering, University of Patras Campus of Rion, Patras 26500, Greece; cantonop@ece.upatras.gr (C.A.); koubias@ece.upatras.gr (S.K.); tsstoyanova@ece.upatras.gr (T.S.); tuliatos@ceid.upatras.gr (G.T.); 2 Telecommunication Systems Institute, TUC Campus, Kounoupidiana, Chania 73100 Greece; as_katerina@yahoo.gr (K.A.); vsam@softnet.tuc.gr (V.S.); jvlahou@gmail.com (G.V.); 3 INTECS S.p.A., Via Giacomo Peroni 130, Rome 00131, Italy; sarah.chiti@intecs.it (S.C.); luca.donofrio@intecs.it (L.D.); simone.gianfranceschi@intecs.it (S.G.); angela.valvo@intecs.it (A.V.); 4 Centro de Electrónica Industrial, Universidad Politécnica de Madrid, C/José Gutiérrez Abascal, 2, Madrid 28006, Spain; danping.he@upm.es (D.H.); gabriel.mujica@upm.es (G.M.); jorge.portilla@upm.es (J.P.); teresa.riesgo@upm.es (T.R.); 5 Università degli Studi di Napoli Federico II, Dip. di Ingegneria Elettrica e delle Tecnologie dell’Informazione, via Claudio 21, Napoli 80125, Italy; iodice@unina.it (A.I.); daniele.riccio@unina.it (D.R.); ruello@unina.it (G.R.); 6 Industrial Systems Institute—ISI/R.C. “ATHENA”, PSP Bldg., Stadiou Strt., Platani, Patras 26504, Greece; koulamas@isi.gr (C.K.); papadopoulos@isi.gr (G.P.); 7 Politecnico di Torino, dip. Elettronica e Telecomunicazioni, corso Duca degli Abruzzi 24, Torino 10129, Italy; luciano.lavagno@polito.it; 8 Métodos y Tecnología de Sistemas y Procesos S.L. C/Santa Leonor 65, Edif. C, Pl.4ª, Madrid 28037, Spain; lredondo@mtp.es (L.R.); daniel.rodriguez@mtp.es (D.R.)

**Keywords:** wireless sensor networks, simulation, Internet of Things, design flow, assisted deployment

## Abstract

In this article we present the main results obtained in the ARTEMIS-JU WSN-DPCM project between October 2011 and September 2015. The first objective of the project was the development of an integrated toolset for Wireless sensor networks (WSN) application planning, development, commissioning and maintenance, which aims to support application domain experts, with limited WSN expertise, to efficiently develop WSN applications from planning to lifetime maintenance. The toolset is made of three main tools: one for planning, one for application development and simulation (which can include hardware nodes), and one for network commissioning and lifetime maintenance. The tools are integrated in a single platform which promotes software reuse by automatically selecting suitable library components for application synthesis and the abstraction of the underlying architecture through the use of a middleware layer. The second objective of the project was to test the effectiveness of the toolset for the development of two case studies in different domains, one for detecting the occupancy state of parking lots and one for monitoring air concentration of harmful gasses near an industrial site.

## 1. Introduction

Wireless sensor networks (WSN) are employed in a wide range of applications and application domains, and are the subject of considerable research efforts and technological advances to expand their applicability. WSNs were among the enabling technologies of the Internet of Things (IoT) paradigm since it was coined more than 15 years ago [1], and consolidated and expanded their application domains as the IoT paradigm evolved.

However, the spread of WSN-based solutions is still limited by several factors, such as technical complexity, perceived reduced reliability and overall cost. The development of efficient WSN applications requires different and complementary views, but competency separation between the typical stakeholders and the engineering disciplines concurring in the WSN domain is not yet mature, which leads to significant inefficiencies. For instance, WSN design and planning can be effort- and risk-intensive, because they require detailed consideration of connectivity, coverage, cost, network longevity, and service quality. In addition, predicting WSN performance before its real deployment is very challenging, which leads to costly trial-and-error procedures.

**Figure 1:**
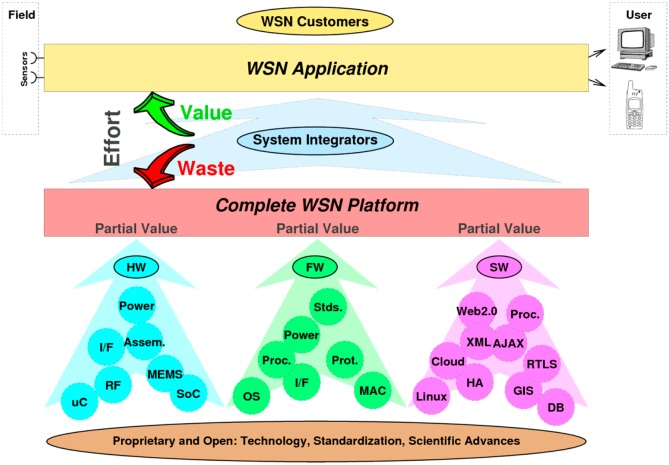
WSN-DPCM toolset helps system integrators to focus on customer requirements by hiding most structural and implementation details of WSN platform components.

The aim of the ARTEMIS-JU funded project “WSN Deployment, Planning, and Commissioning & Maintenance” (WSN-DPCM) presented in this article was to build an integrated platform to support the system integrators to design, develop, test, deploy and maintain WSN applications. The project acknowledged from the beginning that the technical work of system integrators relies heavily on existing hardware and software components and should focus on application-specific parts, as shown in Figure 1.

Thus the primary design objectives of the WSN-DPCM platform were:

- A modular design with components that perform well-defined tasks and communicate through well-defined interfaces and data models, in order to simplify the maintenance and evolution of the platform in line with the fast evolution pace of WSN research and technology;
- To be accessible to application domain experts while allowing experienced developers to optimize the designs as much as possible;
- To reuse open or closed source IP blocks in order to increase the reliability of WSN projects, to reduce the development effort, and to foster commercial exploitation of the platform;
- To allow the developers to focus on application-specific requirements and implementation of the WSN application by reducing the effort needed to select and adapt the existing components to the application, as well as to reduce the effort to plan, commission, troubleshoot and maintain the WSN during its lifetime.

In order to meet these aims, the WSN-DPCM platform includes several useful capabilities, such as radio frequency (RF) simulation, network simulation with hardware-in-the-loop (HiL) capabilities, topology optimization, automatic software synthesis, monitoring, debugging, as described in the rest of the article.

The effectiveness of the WSN-DPCM toolset is tested in two case studies in different domains. The first is a WSN-based system to monitor the occupancy state of parking lots, of which a prototype has been tested in the parking of the Universidad Politécnica de Madrid (UPM), Spain [2,3]. The second case study is a WSN-based solution for monitoring air concentration of harmful gases near an industrial site, of which a prototype has been tested at the INTECS premises near Pisa, Italy [4,5]. Note that both case studies refer to outdoor deployment scenarios. In fact, the proposed toolkit is primarily intended for outdoor WSN applications especially because the ray tracing-based RF simulation is effective to simulate outdoor electromagnetic field propagation. For indoor environments a simple heuristic path loss one-slope model may be used, but with significantly lower prediction accuracy [6].

The rest of the paper is organized as follows: Section 2 briefly reviews the state of the art. Section 3 presents the WSN-DPCM toolset architecture and the typical application design flow. Section 4 details the main tools of the platform. In Section 5 the two use cases of the project are presented, and concluding considerations are made in Section 6.

## 2. Related Work

The main advancement of the WSN-DPCM platform with respect to the state of the art is that it provides a complete integrated toolset for WSN design and management, which is a highly desired feature [7]. Existing WSN frameworks such as ProFuN TG [8] or GOAT [9] provide a full set of tools to support network simulation, application-logic design and network monitoring. However, their features and scope are focused on specific segments of network development and maintenance, and do not aggregate or enable support for the whole WSN tool chain, from development to deployment and maintenance.

Almost every WSN framework and operating system provides integrated simulation support. Recent surveys of simulation tools for WSN [10,11,12,13] list dozens of tools, including instruction-level simulators for popular microprocessor families, RF connectivity simulators, mobility simulators, protocol-level simulators, *etc*. Operating system-based simulators, like TOSSIM [14] and COOJA [15], provide good support for code written for the respective platforms, and are often coupled with hardware-level emulators for popular architectures, for instruction-level simulation. Such tools are best suited to develop and test low-level components, such as routing protocols. Several of the available tools, such as Castalia [16], MiXiM [17] and NesCT [18] are extensions built on top of the general-purpose discrete-event simulator OMNeT++ [19]. These tools provide higher-level abstraction of the WSN and in general can scale to larger networks and longer simulated periods.

An important original feature of the WSN-DPCM toolset is a site-specific RF simulation using a new ray tracing-based RF simulator that is customized for WSN applications and considers in detail the surrounding environment and main obstacles. Many wireless network simulation tools, such as OMNeT++ [19] and TOSSIM [14], currently rely on very simple, heuristic propagation models that do not account for the details of the surrounding environment. On the other hand, the electromagnetic propagation prediction tools that account for complex outdoor environment [20,21,22,23] are tailored for radio and television broadcasting, cellular telephony systems or Wi-Fi, and have not been employed for WSN planning.

Several high-level programming abstractions and design automation have been proposed to improve application development productivity. Shimizu *et al.* [24] propose application decomposition at network-, group- and node-level and a Domain-Specific Modeling Language optimized for each level. Application development using this model is restricted mainly to optimization of several parameters. REMORA [25] is an advanced component-based design framework that uses C-like behavioral models wrapped in an XML-based abstraction of, e.g., services, interfaces and events. The run-time overhead is low, but the approach cannot handle arbitrary behavioral code or hardware specifications. Song *et al.* [26] demonstrated a model-based hardware-software co-design framework based on widely used tools like Mathworks Simulink [27] and Stateflow [28]. They provide a high-level graphical application entry based on abstract concurrent models, node-level simulation, automatic generation of network simulation models (including hardware-in-the-loop) and implementation models for popular embedded OSs. However, the developer is required to manually define and connect all modules that compose the application.

In HiL testing, the program under test runs on the physical sensor nodes with some assistant middleware. For example, TOSSIM simulates the sensor network by replacing low-level components and introducing a discrete event queue. Developers can test the program before the TinyOS application is deployed. However, TOSSIM cannot reveal if the length of a message is set to be less than its intended size or it cannot reveal problems stemming from the duration of tasks, since the tasks in TOSSIM are executed instantaneously. Moreover, it has not a general mechanism in order to accurately estimate the power consumption of the sensor nodes. In [29] a solution aiming to alleviate the requirement that not all TinyOS programs run on physical nodes is proposed, because that increases costs excessively while being inconvenient, so they allow only one physical node to exist which can be configured to be a neighbor of any virtual node. Thus, the potential faults which cannot be captured by pure simulation testing tools can be captured through the interaction between the physical node and other virtual nodes. Another advantage of their work is that the power consumption of a node in a large WSN can be estimated through the use of low-cost digital multimeters. Some considerations not taken under account on that work include the signal gains that currently are designated by the user and the fact that power consumption estimation may be improved.

In [30] a hybrid simulation framework for WSN application development is proposed, that interconnects a virtual network with a physical network and then allows one to simulate the network as a whole. The authors created a novel model-based hybrid simulation framework for WSN applications, introducing the notion of hybrid simulation. They exploit HiL extensions in order to link some of the simulated WSN nodes with hardware-dependent features. Thus, by using Hy-Sim users can build a hybrid network consisting of virtual and real nodes and then simulate it as a whole. Actually, Hy-Sim supports the existence of purely virtual nodes, of purely physical nodes, but also of partially simulated nodes. The latter can have access to real sensors in order to complete part of their operations. Moreover, Hy-Sim allows nodes to have access to a real channel and even have access to data coming from real sensors.

Although there have been contributions in the literature regarding the optimization of the wireless sensor networks from the point of view of planning and simulation technologies, there is still a shortage of methodologies and frameworks to carry out the in-field deployment, configuration and performance assessment of the overall system application. In [31], the authors proposed an embedded initialization and maintenance mechanism in which a flooding-based synchronization strategy is adopted to configure discovery and expiration time windows into which nodes can perform neighbor search and link assessment. Operational modes are triggered by dedicated points of the network, such as sink and root nodes, from which the mode changing process is realized. In order to enhance the flooding process and wake-up calls, techniques such as Trickle [32] are also considered. Another maintenance system to be embedded into the sensor nodes has been proposed in [33], which intends to provide maintenance services during the operational stage of the WSN by defining system coherency state policies. Such maintenance policies mainly rely on triggering conditions when encountering sensing failures and communication coverage failures, particularly considering protocol coherent states, which are organized as Global view of coherent states (system-wise, such as time synchronization requirements), and Local view of coherent state (node-wise, such as neighborhood awareness). The maintenance engine collects the coherent requirements and states, and then floods the list of protocols for the maintenance service to the network. Other works, such as the one proposed in [34], make use of Deployment Support Networks (DSN), which are wireless network temporarily installed alongside the actual WSN during the deployment process. The main target of this infrastructure is to provide two different radio communication capabilities, one for passive listening of network traffic and one for debugging channel connections through DSN nodes to distribute the gathered information to a nearby sink point, from which the deployment analysis will be performed. Therefore, such a debugging implementation relies on a passive monitoring system based on the deployment of additional network platforms.

## 3. WSN-DPCM Architecture and Design Flow

The WSN-DPCM toolset flow was designed to reduce the application development and deployment effort and the time-to-market for new WSN applications. It enables software-assisted engineering and provides most of its integrated capabilities as services over the Internet.

**Figure 2:**
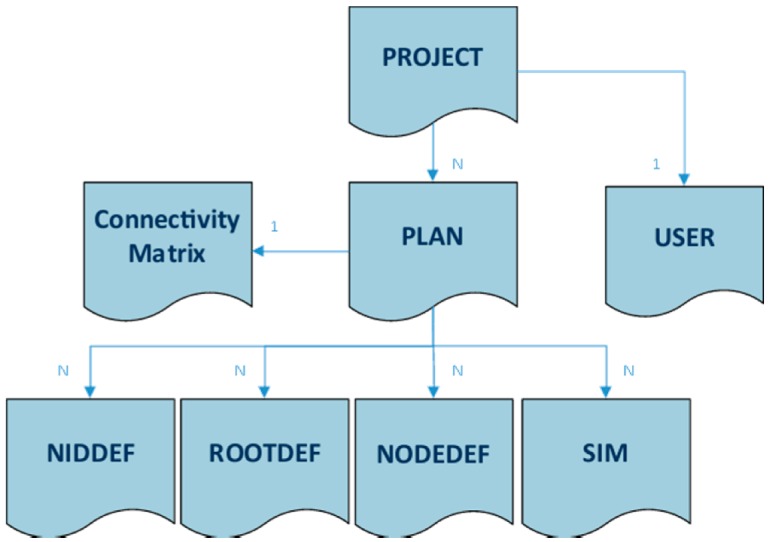
Diagram of the data model hierarchy and the multiplicities among its entities.

Its operation flow is correlated with the DPCM data model, which holds all information items used by the different tools of the Platform as input/output, from the creation of a new Project to online monitoring of the designed WSN network in a real-life environment. Model entities can be created, modified or deleted throughout a toolset session. The hierarchy of the model is shown in Figure 2. It comprises the following main entity types:

- PROJECT—project description and attributes. It includes references to the USER and the list of PLAN entities defined for the Project.
- PLAN—deployment plan definition and attributes. It includes references to SIM entities as a result of the simulations executed over the plan specification, the CONNECTIVITY matrix entity as attachment and all references to the definition entities of the node types considered by a particular deployment.
- USER—contact information and credentials of the project’s user.
- CONNECTIVITY MATRIX—structured file in JSON format that contains the communication properties and transmission values estimated by the simulation of the radio links between each pair of nodes.
- NIDDEF—type definition entity for network interface devices (NID, e.g., gateways). It contains all details that characterize a NID type structurally (type identifier, nature, functional blocks, parameters *etc.*) and functionally (properties, configuration choices, *etc.*). This specification is correlated with the embedded code generated for this particular type of node.
- ROOTDEF—type definition entity for sink node devices, similar to the NIDDEF entity. It can contain node programming software when the code synthesis phase is completed.
- NODEDEF—type definition entity for regular leaf node devices, similar to the NIDDEF and ROOTDEF entities. It can contain node programming software when the code synthesis phase is completed.
- SIM—RF simulation entity that contain its configuration attributes and results for the targeted nodes.

**Figure 3:**
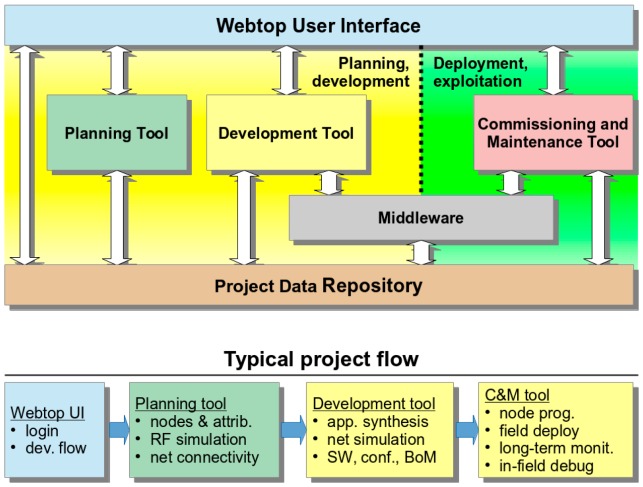
Overview of the WSN-DPCM Toolset communication and typical development flow.

The platform is based on three main tools, the Planning Tool, the Development Tool and the Commissioning & Maintenance Tool, and supporting components, Library of components and the Middleware, each consisting of integrated services that are accessible through a local/web GUI (WebTop Environment) and/or RESTful services (Figure 3).

The WebTop Environment is a web desktop application that requires no local installation and acts as a unique entry point to the toolset through user authentication. It provides the main user interface to the WSN-DPCM toolset, all visualization support needed by its tools and other information, as well as the controls needed to advance through the development flow. As shown in Figure 3, it has control interfaces with each of the main platform tools in order to control their execution (e.g., start, stop, status) and data interfaces with the Project Repository of the platform in order to check the advance status of the various development stages, as reported by the tools.

The Planning Tool (PT) provides the developer with a graphical input interface for the deployment field map, for the definition of WSN node types and their positions in the field, and for other platform tools that need to analyze various aspects of node deployment (e.g., RF propagation, signal strength and coverage strength, optimal topology and connectivity). It has a control interface with the WebTop Environment and a data interface with the Project Repository of the platform to save new projects or retrieve older ones.

An important PT analysis is radio coverage of the nodes based on their positions in the application field, their radio characteristics and field features. This analysis is controlled from the user interface of the Planning Tool. The results are graphically overlaid on the application field map and detailed information is also available in textual form for each node in the field. The tool uses the results of radio simulation to calculate the node connectivity matrix, which is saved in the Project Repository for later use by the Network Simulator within the Development Tool.

The deployment plans designed by the PT tool are live elements that can be adapted and refined using the feedback from simulation analysis or the deployment reports produced by Platform tools and systems. In general, the DPCM toolset considers an iterative approach to solution prototyping using the proposed work flow (Figure 3) through sequences of planning, simulation, synthesis, deployment, monitoring activities, by updating the data models to the new environment and prototype requirements.

The Development Tool (DT) allows the developer to produce the application-specific software to program the WSN nodes and to simulate their behavior in the field using network simulation, either purely in software or including real hardware nodes (hardware-in-the-loop simulation: HiL) to increase result accuracy. HiL is a technique increasingly considered in the development and testing of embedded systems, where the main goal is to test the embedded system itself instead of an abstract model. The complexity of the real plant under control is embodied in the simulation, while the designed system must be tested using realistic simulation conditions in a virtual environment. HiL is a kind of real-time simulation where the input and output signals of the simulator show the time dependent values as they are in the real process.

The tool maintains a control interface with the WebTop Environment over which it can be started and can communicate the completion status for its internal development steps (e.g., application synthesis, network simulation). It has also a data interface with the Project Repository of the platform to save new projects and results (e.g., node application specifications, simulation results) and to retrieve older ones.

As shown in Figure 3, DT interfaces with the Planning Tool mainly to extract the list of requested node types and their features from the network plans saved by PT in the Project Repository or the node connectivity matrix. For each node type, DT automatically creates a development project skeleton for which it provides several node-level application development flows. For application domain experts, the tool offers a guided, graphical flow based on state charts, which requires just basic programming skills. Nevertheless, experienced programmers can use other input methods, which allow them a finer control over node program development. Regardless of the input method of the application-specific business logic, DT provides automatic synthesis of the necessary software and hardware to support the application requirements using a library of hardware and software components, with or without the support of an embedded operating system or middleware. The synthesis process aims to hide most implementation details that are not explicitly addressed by the application specification (e.g., some low-level sensor implementations, OS tasks, interfaces) and to facilitate software reuse, allowing this way the developers to focus on application-specific issues.

The Commissioning and Maintenance Tool (CMT) assists the developer in the preparation of the nodes for field deployment, during the actual field deployment operations, and for monitoring and debugging after deployment for long-term maintenance. 

For node preparation for in-field deployment, the CMT uses the programming images and configurations saved in the Project Repository by the Development Tool for all nodes, and the node type and field position saved in the repository by the Planning Tool. Once deployed in the field and activated, the actual operation conditions of the nodes are collected by the CMT and can be compared with the results of the radio and network simulations saved in the Project Repository to correct and optimize various network parameters. The CMT is in sync with the deployment specification, being able to reconfigure itself automatically whenever a new Plan is generated or modified, or the firmware images are changed, reconfigured or regenerated.

The Middleware (MW) provides an abstraction layer for node resources, software and network services, and communication capabilities within the network. This layer allows to write implementation-independent applications to increase software reuse and development productivity. It can interface with the Development Tool to support application synthesis and its embedded support is used at run-time by the CMT to monitor the operation of the network.

A typical application development using the WSN-DPCM platform starts by authenticating the developer with the platform, through the Webtop-based UI (see the lower part of Figure 3). The UI helps the developer to follow the platform development flow by activating only the commands that meet all conditions to be executed. For instance, at the begin of a new project only the Planning Tool can be activated to allow the developer to input, e.g., the deployment field map, the definition of the node types and their positions in the application field. Afterwards, the developer can run a simulation of the RF propagation to estimate the number and quality of RF communication links. If these are not satisfactory, the developer can revisit the map and node properties and make the necessary changes to fulfill all quality criteria.

Once a field plan is approved and saved in the Project Repository (PR), the UI activates the next development step in the flow, the Development Tool. This tool collects node data and network connectivity from the plan. Node types are automatically extracted and for each of them the tool creates a project template that can be used by the developer to subsequently specify all node characteristics and the application it needs to run. For each of these projects, the developer can run the application synthesis engine whose output are software for node programming, hardware specification and network simulation model. The latter can be used together with the connectivity matrix generated by the RF simulator in the Planning Tool to perform pure software network simulation or mixed, with hardware nodes in the loop. If the synthesis or simulation results are not suitable, the developer can iteratively change the node specifications or the simulation parameters within the Development Tool, or can go back to the Planning Tool, e.g., to change network composition or topology.

When node development is completed and saved in the Project Repository, the UI enables the next development step, the Commissioning and Maintenance Tool. This tool extracts the node programming software, configurations and hardware specifications from the repository and use them to program deployment-ready nodes. Moreover, it uses the field map and node locations from the plan generated by the Planning Tool to program the CMT interface for long-term network monitoring and to program the Hand-held devices that will be used during network deployment and for in-field debugging. If the data collected in the field are not satisfactory, the developer can go back to the Planning or Development tools to make the necessary amendments to the network plan, or node or application specifications.

If the environment (*i.e.*, the scenario in which sensors are deployed) changes, the RF simulation and the connectivity matrix can be run again for all nodes in the updated scene. If only some nodes changed, the simulation can be run just for those nodes to save computation time (see Section 4.1.2). If the connectivity matrix changes, the network simulation of the Development Tool may need to be run again to check the suitability of the new network. However, if new node types are added or either the node or application specifications change, then it is necessary to re-run the synthesis engine of the Development Tool to update the simulation models, the node configuration and programming code, and the hardware configuration.

The Commissioning and Maintenance Tool should also be updated with the new plan, software and hardware components in order to correctly interface and interpret the field data during deployment and the long-term network exploitation.

## 4. Main Tools of the WSN-DPCM Platform

The operation of the three main tools of the WSN-DPCM platform (Planning Tool, Development Tool, and Commissioning & Maintenance Tool, see Figure 3) is controlled from a web-based platform, while the operation of the underlying hardware and software components and services is abstracted through a Middleware component, also developed within the project.

### 4.1. Tool for WSN Application Planning and RF Simulation

The Planning Tool consists of:

- An input interface that displays the map of the deployment field and allows WSN node selection and positioning,
- A set of functional components for RF propagation simulation and node connectivity prediction.

Different types of nodes can be selected from those available in the repository and/or new ones can be defined by the developer. Once selected, the nodes can be placed on the map with a click on the desired position. The other functional components integrated in the PT are described in the following.

#### 4.1.1. 3D Building Reconstruction

To improve the simulation accuracy, 3D reconstruction of physical environment is very desirable. When environment description is not available or is too expensive, the developers need to build it by themselves.

However, for many radio propagation experts the preparation of the environment model is more challenging than analyzing the radio performance. Besides, most research results presented in academic conferences reuse the same environment to analyze their work and cannot test the robustness and accuracy for different environments.

**Figure 4:**
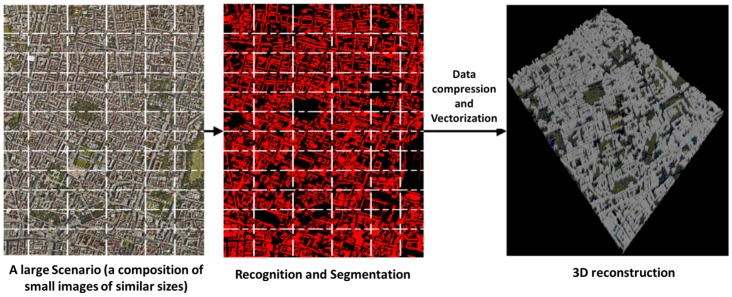
Work flow of the 3D outdoor environment reconstruction.

For this purpose, the WSN-DPCM toolset includes a functional component that can automatically reconstruct the third dimension from 2D images (Figure 4). It extends the algorithm proposed in [35] and uses sub-sampling and random feature selection techniques for iterative learning from images. The estimated confidence value of each class for each pixel can be reinterpreted as a probability distribution using soft max transformation to calculate texture layout potentials.

The work flow of the method is shown in Figure 4. Meaningful object classes are recognized through a machine learning mechanism, which consists of a training phase followed by an evaluation phase. The training phase uses an image database through which the features of images are learned and discriminated for the concerned types of objects. After an image is properly recognized, the algorithm automatically assigns different volumetric information according to scale ratio and empirical material for each object, as it can be seen in Figure 4.

#### 4.1.2. RF Simulation and Connectivity Matrix

This component has two main functions within the WSN-DPCM platform: to allow the developer to check node connectivity for a given placement in the application field and to generate the input data for the topology reduction and network simulation components of the WSN-DPCM platform. Its main features have been recently presented at a conference [36] and are here better detailed.

The component calculates the received signal strength for each node when each of the other nodes in the network is transmitting (one at a time) using an electromagnetic solver, or RF tool. It receives as input a digital description of the application scene and the technical specifications of the transmitting antenna. The former is provided using a file in Keyhole Markup Language (KML) format that describes the buildings, and a raster file that describes the topography of the terrain, *i.e.*, the Digital Terrain Model (DTM), if available. The format employed for building description simplifies data import from various sources, such as local authorities, Google Earth or Google Maps, or reconstructions from aerial photography using the component described in Section 4.1.1. Relative permittivity and conductivity of building walls and terrain can be added to improve simulation accuracy, otherwise the simulator uses sensible defaults based on area type (historical, residential, business district). The transmitting antenna is described by its position, radiated power, pointing direction, radiation diagram and polarization.

The solver is based on a 3D space analysis similar to that described in [22,23] using a ray tracing algorithm that considers direct, reflected and diffracted rays. In particular, reflections are computed using Geometrical Optics (GO) and diffractions are evaluated using the Uniform Theory of Diffraction (UTD). The user may also select a “fast mode” that only accounts for the direct and reflected rays, to avoid most computational load which is due to diffracted rays.

**Figure 5:**
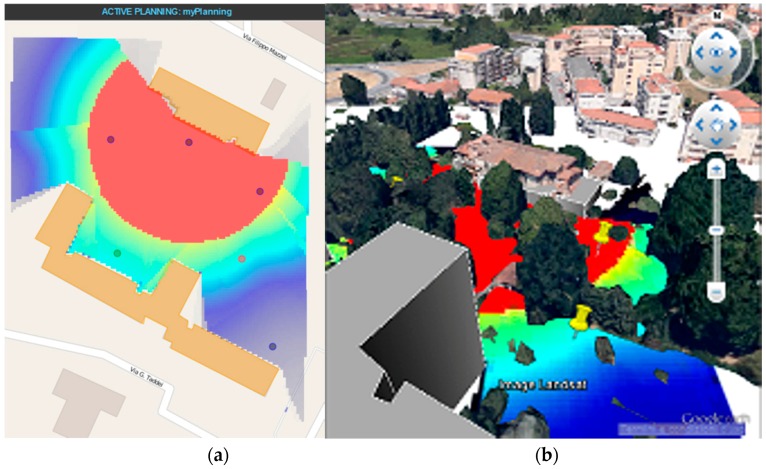
2D graphical display of the computed field radiated by one of the nodes (**a**) that can be superimposed to Google Earth image of the area (**b**).

**Figure 6:**
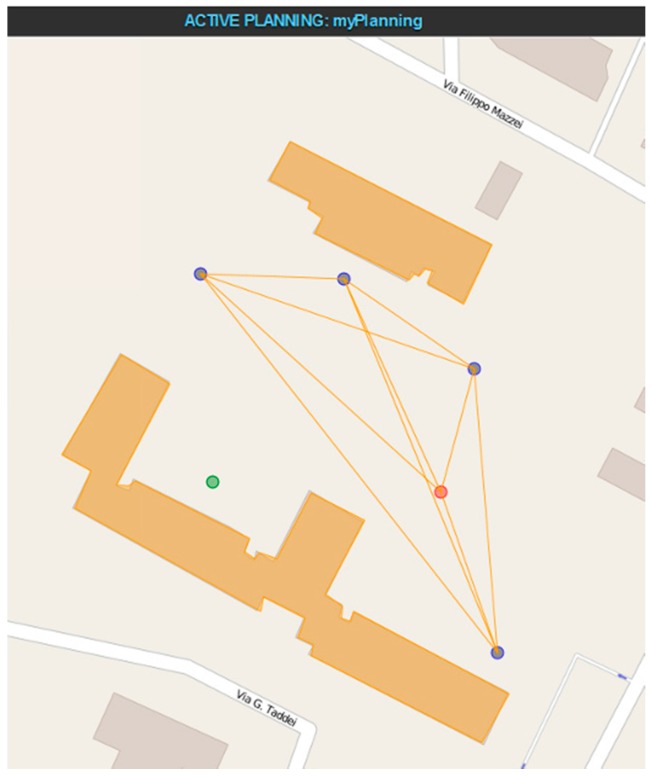
Graphical representation of the connectivity matrix.

The resulting electromagnetic field is provided on regular 2D grids (“layers”) located on surfaces at well-defined heights above the ground (or above the roof, if the grid point is on top of a building). Accordingly, a 3D map is obtained, which shows the field levels produced by the transmitting antenna in the considered scene. The map is stored in geotiff format; it can be displayed by the WebTop user interface (GUI) and optionally superimposed on a Google Earth image (as shown in Figure 5), and/or it can be passed to the connectivity matrix module. The latter, based on the field maps produced by the electromagnetic solver, computes and stores the field values radiated by each antenna (node) at the locations of all other antennas (nodes) in a “connectivity matrix”. This matrix can be easily converted in a binary matrix if receiver field threshold levels are defined. Non-zero elements of the matrix can be displayed by the GUI as arcs connecting the corresponding nodes, as shown in Figure 6.

A typical use of this tool within the WSN-DPCM toolset starts from a 3D description of the scene in which the developer places the motes based on the sensing requirements of the application. Then, the developer selects a propagation model (fast, for quick but less accurate simulation, or standard, for slower but more accurate simulation). The RF tool calls the solver and connectivity matrix modules to compute the field levels generated by the transmitter of each node in the whole scene and uses them to generate the connectivity matrix, as explained before. Based on these results, the developer may iteratively refine node positions, optionally using the topology reduction tool (see Section 4.1.3). Once the results are acceptable, the matrix is stored in the Platform Repository where it can be retrieved by the network simulator (see Section 4.2.1) for further consistency and suitability checks. If necessary, the whole procedure may be repeated.

We analyzed in detail the processing time and scalability of the algorithms both theoretically and experimentally [37]. Computation time increases linearly with the number of nodes and with the cube of the linear size of the scene. Fast mode computation time is one order of magnitude smaller than standard mode. For example, fast mode processing of a scene of 1 km linear size ranges from 30 s for five nodes to 50 min for 500 nodes on a four core Intel(R) Xeon(R) CPU E5440 @ 2.83 GHz with 3 GB RAM. In all cases, the computational load to generate the connectivity matrix is only a very small fraction of the RF simulation, less than 0.2%.

#### 4.1.3. Topology Reduction

The usefulness and added value of the PT towards network deployment definition is significantly enhanced through a set of novel algorithms developed in the context of the WSN-DPCM project that allow the developer to evaluate network connectivity and propose alternative connectivity scenarios. It is noted that respective algorithms developed are based on RSSI values provided by the RF simulator or real measurements acquired from a network deployment. To the best of the authors’ knowledge, it is the first time that well-known graph theory algorithms have been utilized to extract useful information about a network topology as well as to attempt to offer alternative topologies.

##### Network Connectivity Evaluation

Based on the connectivity matrix, these algorithms exploit graph theory in a novel approach that allows the developer to identify critical characteristics of the network (assuming a specific topology scenario) which can have a great impact on network performance and robustness. This effectively allows the developer to modify the deployment accordingly until the topology satisfies all requirements. Specifically, through these algorithms the developer is notified if the network is segmented (*i.e.*, there is a node or group(s) of nodes that cannot communicate with some other group(s) of nodes) or regarding the existence of a critical node (*i.e.*, a node that segments the network if it fails). Also, performance-wise valuable indications are offered regarding the number of hops that separate each node from the nearest (or the assigned) gateway. Furthermore, awareness on possible congested areas can be deduced through the number of neighbors of a specific node.

In order to achieve the aforementioned objectives, we use two approaches. One is based on Algebraic Graph Theory (AGT) to represent network topology as a graph and analyze it using linear algebra and matrix theory [38]. The other approach is based on Depth First Search (DFS) [39] to obtain accurate results on connectivity evaluation, on checking for network segmentation and for critical nodes. The developed algorithms are presented and evaluated in [40]. In this paper, the topology evaluation algorithms are tested in various network topology scenarios in MATLAB and manage to identify in all cases: (1) if the network is fully connected or not; (2) if there are critical nodes; (3) for a partitioned network which nodes are in each partition; and (4) if there are isolated nodes.

**Figure 7:**
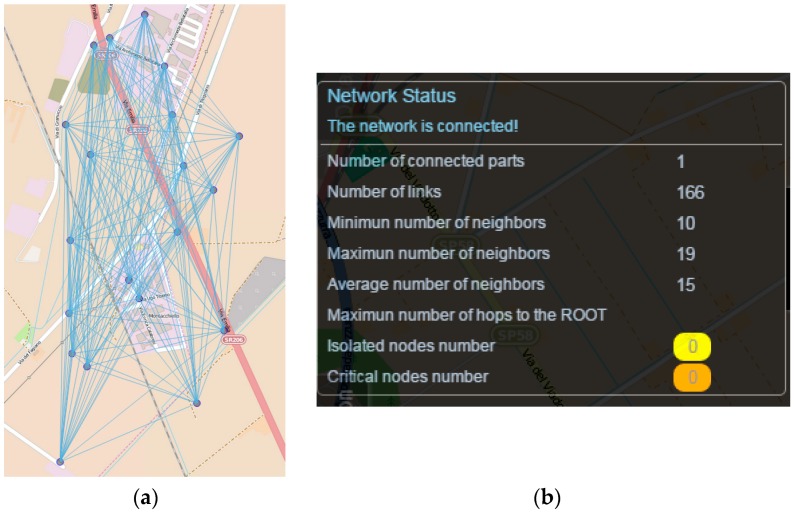
WSN-DPCM Integrated Network Evaluation Algorithms results: graphical (**a**) and statistics (**b**).

The developed tools have been fully integrated in WSN-DPCM Planning Tool, as shown in Figure 7, in which a 2 km^2^ area has been covered using 20 nodes. In this case, the analysis results show that the network is fully connected and has no critical nodes. Moreover, the average number of neighbors is indicated (15 in this case), which, in combination with data traffic patterns, can reveal possible congestion hot spots in the network. Also, all nodes are able to directly communicate with the gateway, since no indication appears under the “Maximum number of hops to the ROOT” metric. This case will be utilized in the following as a reference scenario to present approaches able to suggest alternative topologies.

##### Alternative Reduced Topology Indication

To further enhance PT features, a more experienced developer can set specific parameters or requirements. In this respect, another group of algorithms was designed, implemented and integrated in the context of the WSN-DPCM project that is able to suggest alternative routes or parameters in order to meet specific requirements. All the above form a group of valuable and easy to use tools that are missing in the state of the art environments.

Starting from the initial network that is usually created with maximum transmission power, we perform link reduction by targeting the following parameters: predefined number of neighbors, predefined n-connected topology and transmission power reduction. These approaches and mainly their relative integration effort in WSN-DPCM Planning Tool are described next. It is noted that the core algorithms have been presented and evaluated in [41] under various cases of controllable network topological scenarios in MATLAB.

K-ROUTE: MST-based link reduction algorithm [38]. Given a connected, undirected graph, a spanning tree of that graph is a sub-graph comprising a tree that connects all vertices. A graph can have many different spanning trees. We can also assign a weight to each edge, which is a number representing how unfavorable this edge is, and use this to compute the sum of the weights of the edges in that spanning tree. A minimum spanning tree (MST), or minimum weight spanning tree, is then a spanning tree with weight less than or equal to the weight of every other spanning tree. If each edge has a distinct weight, then the MST is unique. From a more abstract point of view, any undirected graph has a minimum spanning forest, which is a union of minimum spanning trees for its connected components.

**Figure 8:**
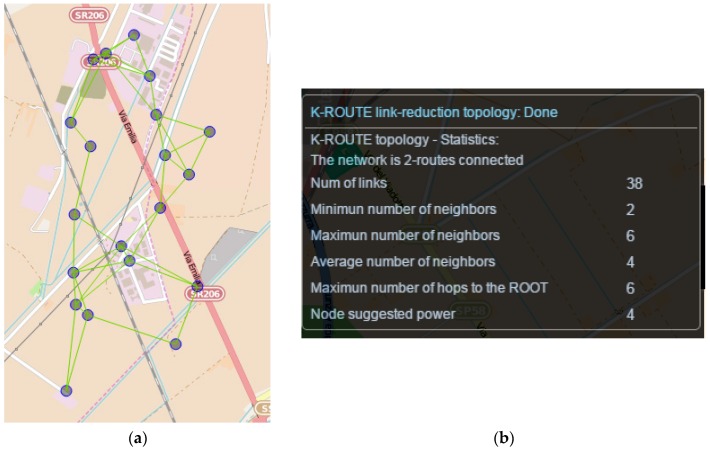
WSN-DPCM K-Route Link Reduction Integrated Algorithm (power in dBm): graphical results (**a**) and statistics (**b**).

Figure 8 shows the effect of the respective integrated algorithm to the same topology scenario assuming a requirement of 2-route connected graph. So effectively, and with respect to Figure 7 topology, in this case initially the optimal minimum spanning tree is identified (*i.e.*, the MST with the minimum cost edges which represents the most robust connections). Then, the identified MST is excluded and the second best MST topology is identified. Finally, the two identified MST topologies are merged to provide the final alternative topology. As indicated, the number of links and average neighbors are significantly reduced (from 166 to 38 and from 15 to 4), which can enhance networks efficiency and resource conservation. However, as expected, the number of hops to ROOT nodes is also increased (from 0 to 6), which indicates the need for multi-hop communication and routing protocols.

N-NEIGHB: Neighbor-based link reduction algorithm. The algorithm attempts to ensure a specific number of neighbors. Actually, the minimum number of nodes is bounded, hence for some nodes the number of neighbors could be slightly increased.

The process starts from the node with the smallest number of neighbors. The first n-neighbors with the strongest RSS are added in the new neighbor table for this node. The algorithm continues by processing all nodes in ascending order of their number of neighbors. Before adding a new neighbor to the table, its number of neighbors is checked. If this number is equal or larger than required, this neighbor is discarded and the next neighbor in the list is evaluated. If all neighbors of a node have more than the required number of neighbors, but this node has not reached the required number of neighbors, then the first n-neighbors with the strongest RSS are added in the nodes neighbor table even through these neighbors exceed the required number of neighbors.

**Figure 9:**
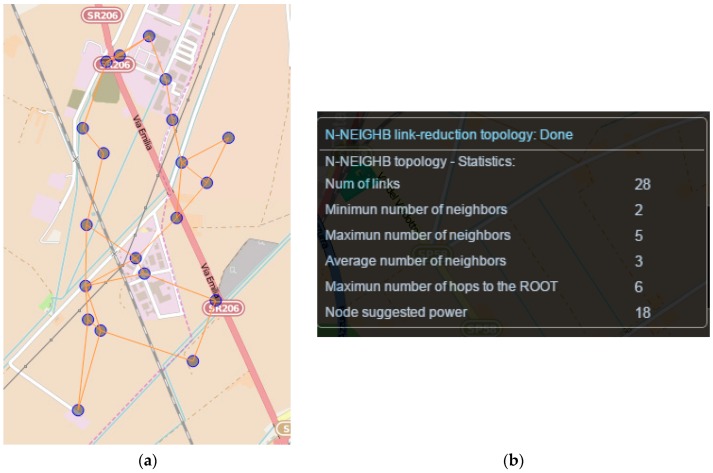
WSN-DPCM N-Neighbor Link Reduction Integrated Algorithm (power in dBm): graphical results (**a**) and statistics (**b**).

Figure 9 shows an example integrated in WSN-DPCM planning tool. Comparing the extracted metrics with the respective measurements shown in Figure 7, it is shown that the proposed algorithm enables the developer to find the optimum trade-off between the number of neighbors/links and the number of hops to the ROOT node.

TxPR: Transmission power-based link reduction algorithm. Instead of transmitting with the maximal power, the nodes can collaboratively reduce their transmitted power and define network topology by forming neighbor relations with specific criteria. In a homogeneous network (all sensor nodes are similar), the most important centralized topology construction algorithms that build the reduced topology by controlling the transmission power of the nodes are those who solve the two well-known problems: the Critical Transmission Range (CTR) problem and the Range Assignment (RA) problem. CTR finds the minimal communication range for all nodes in the network while preserving network connectivity and RA finds the optimal transmission power for each individual node. For efficiency reasons, the implementations have been merged and integrated in the previous two algorithms, K-Route and N-Neighbor. The result is indicated in the metrics next to “Node Suggested Power” label. Therefore, the developer is informed on link reduction possibilities and energy conservation effects of each option, to help finding the best deployment for a specific scenario and requirements.

### 4.2. Tool for WSN Application Development and Simulation

The main functions of the Development Tool within the WSN-DPCM platform are:

- To support the design of top-level application-specific behavior and properties at node level;
- To synthesize node hardware and software using the top-level application specifications and library components;
- To generate simulation code and perform network simulations;
- To generate and compile node programming code;
- To generate application-specific configuration for nodes and for the Commissioning and Maintenance Tool.

**Figure 10:**
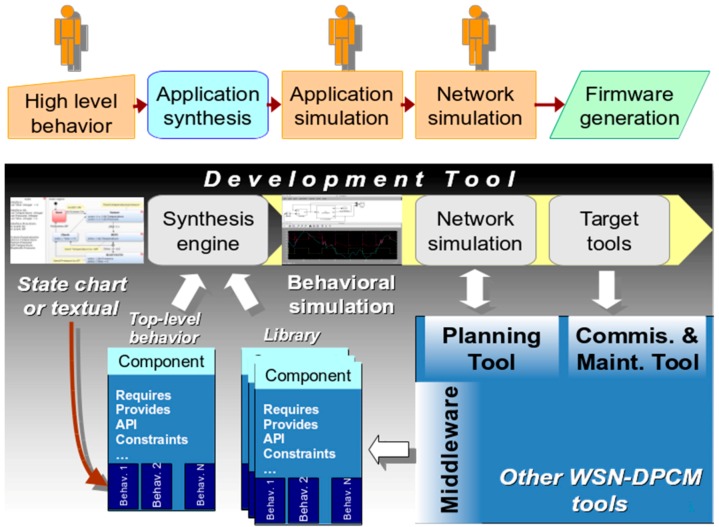
Main stages of semi-automated Development Tool flow.

Figure 10 shows the typical Development Tool flow. It starts with the input of the application-specific behavior, which can range from manual input of source code to automated code generation from model-based design (MBD) abstractions, such as state charts [42], metaprogramming approaches [43], UML-based or *ad-hoc* high-level modeling flows [24,44,45].

Behavioral input is captured in a specific view of the top-level component, which also includes all metadata needed by the subsequent phase of the flow, the automated hardware-software system synthesis. The same component format is used for all library components and can include more than one view, e.g., behavioral source code or binary, simulation models for various abstraction levels and simulation environments. Regardless of their format and abstraction level, these views are always handled as black boxes by the synthesis engine, which relies only on the metadata for its operation.

For the WSN-DPCM flow we developed a project generator that uses the network defined by the WSN planner using the Planning Tool to extract the distinct node types. For each type, the generator creates a synthesis project skeleton, which includes all node specifications already present in network planning and allows the developer to add behavioral code (e.g., using a high-level state chart interface) and other constraints.

These are saved in the format of top-level components that drive the second phase of the flow shown in Figure 10, which is fully automated. In this phase, the synthesis engine of the tool receives as input the top-level component(s) and processes their metadata (such as requires, provides, conflicts). These properties are the starting point for the synthesis process, which iteratively attempts to find all subsets of its library of components that do not have unsatisfied requirements and, at the same time, satisfy all constraints of the top-level and all other components included in the solution.

**Figure 11:**
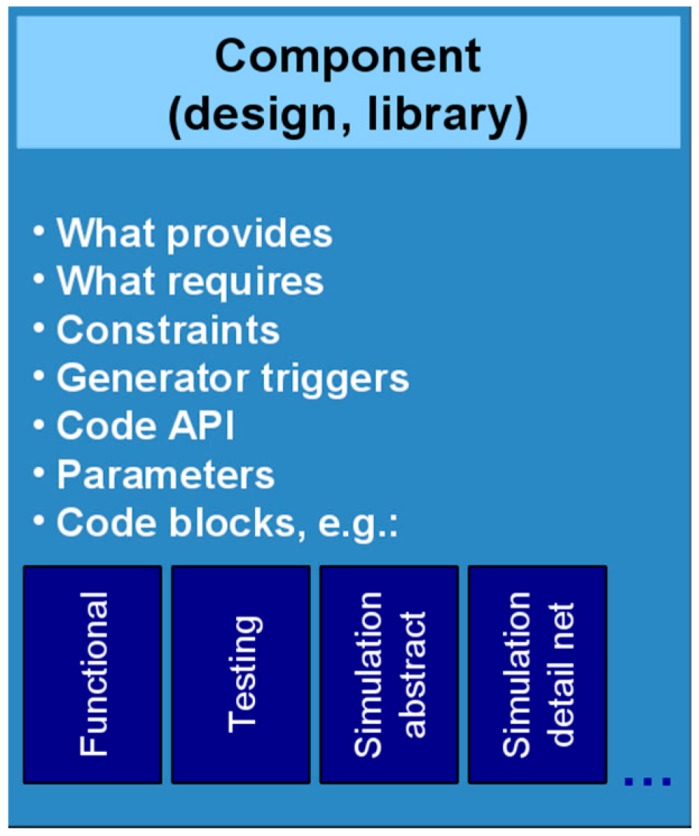
Top-level application specification component and library component structure includes different views (darker on the bottom and handled as black boxes by the synthesis engine) and metadata expressing component requirements and capabilities.

Library components (Figure 11) are central for system synthesis engine operation in order to:

- Store the behavior and requirements of the node-level WSN application modeled as a top-level component by the developer;
- Define the library blocks that can be instantiated by the system synthesis tool;
- Interface with OS or middleware services when necessary;
- Provide simulation models, at different levels of abstraction;
- Provide the target code that implements their behavior to program the nodes;
- Provide code generators that can be run during system synthesis to check if the component can be configured for the specific requirements of the current solution, or to build specialized code stubs based on the actual parameters of the current solution (e.g., for API translation and component code configuration).

**Figure 12:**
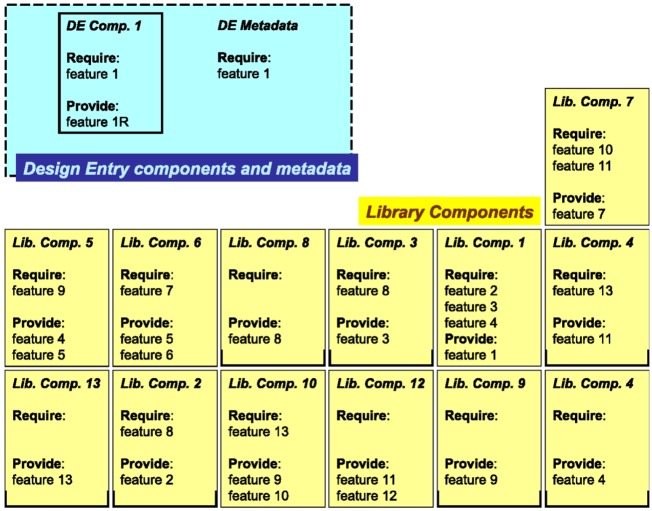
Simplified example of metadata and synthesis.

Figure 12 shows a simplified representation of just a few metadata properties for both library components (bottom) and the top-level specification component (top-left). Metadata specifications of the top-level component drive the beginning of the system synthesis process. As other library components are instantiated in the partial solution, their metadata requirements will drive future searches of the tool together with the still unsatisfied specifications of the top-level component. For the whole duration of the synthesis, the top-level component and its metadata are considered mandatory, while the library components can be instantiated and removed from solution as needed to satisfy design requirements.

A solution is found when all component requirements are satisfied. Once found, the solution is saved along with the actual values for all configuration parameters and can be further examined or modified by the developer. The solver will resume the search by progressively removing the components from the current solution and replacing them with alternatives, if any.

For each generated solution, the synthesis tool can create simulation projects, as shown in the next steps of the flow in Figure 10. They are set up to run on external simulators and can be at various level of abstraction. Basically, this is done by extracting and configuring into the simulation projects the suitable simulation views of the components of the solution.

Besides the behavioral models, the components and the constraints of the solution can include a bill of materials (e.g., compatible nodes, RF and transducer characteristics) or software dependencies on specific compilation toolchains or underlying operating system (OS) features (if any).

Finally, the same mechanism is used to generate projects that can be compiled with the target tools to create WSN node programs. These projects are typically generated in the format expected by the target tools, which most often is a make-based project.

The solutions generated by the tool can be used as they are, or the developer can optimize them either by changing the specification and rerunning the synthesis, or by manually editing the generated projects. Either way, the developer can use simulations to validate and evaluate solutions and improvements.

Figure 10 also shows the typical interfaces of the Development Tool with the Middleware, the Planning Tool and the Commissioning and Maintenance Tool within the WSN-DPCM platform. The Development Tool is designed modular, reuses existing projects as much as possible and provides well-defined clean interfaces to simplify the update or replacement of its components, or the addition of new ones to support specialized development flows.

#### 4.2.1. Network Simulation

The goal of the Network Simulator component is to design and execute whole-network simulations of WSN applications, with minimum developer effort. It supports the estimation of network-wide performance metrics via discrete-event simulation (e.g., end-to-end network latency), at the level of local network neighborhood (e.g., packet loss at link level), or at the level of individual nodes (e.g., per-node power consumption or buffer overruns).

The tool plays an integral part in at least two locations in the design flow. During network design, it can assist decisions regarding node number and placement in the deployment field, for different WSN topologies. During node-level application development, the tool can provide guidance for selection and parameterization of alternative node-level functions, for example the choice of routing protocol and related parameters, or tuning of periodic tasks. Additionally, the tool supports Hardware-in-the-Loop (HiL) simulation.

Creating a network simulation is more than executing a WSN system model. There are at least two additional tasks, which can require non-negligible effort for large networks:

- In order to test a WSN system under different scenaria, the user must provide a simulation of the “environment”, which includes generation of values sensed at the motes, and exceptional events, such as random node failures.
- A simulation may produce copious amounts of output, which requires statistical analysis and graphical visualization, in order to be useful to the user.

In WSN-DPCM, our aim was to improve on the state of the art in network simulation, well beyond previous work, along the following lines:

- Accuracy: it is important to use high-quality, accurate simulation models and algorithms, in order to trust the simulation results.
- Versatility: our tool should allow complex environment simulation, sophisticated result post-processing, seamless integration with HiL, *etc*. In contrast, many frameworks offer restricted facilities in this respect.
- Correctness: the user should trust that the results reflect the actual behavior of the simulated WSN, and are not due to incorrect simulation setup.
- Reduced effort: the effort needed to create a simulation should be minimal.
- Smooth learning curve: learning to use the network simulator should not require extensive effort.

To achieve high accuracy, we employed the Castalia library of components, which runs on the OMNeT++ simulation engine. Castalia is a mature, well-tested and widely used library, regarded as one of the best available. We adapted Castalia’s basic models to suit the requirements of WSN-DPCM and we augmented its model library with additional models for routing and radio devices.

Castalia is highly versatile, allowing arbitrary environment models to be added to the simulation, and providing detailed output. However, exposing the WSN-DPCM user to Castalia would have severe drawbacks, in terms of correctness, effort and learning curve.

- Castalia’s simulation models are very detailed; however, this comes at the price of having to configure hundreds, or even thousands of model parameters in a large simulation.
- The coding of the environment simulation must be done in C++, which is problematic, because (a) it may be hard to debug and (b) requires familiarity with the Castalia and OMNeT++ API, steepening the learning curve.

For these reasons, the underlying Castalia/OMNeT++ implementation is completely opaque to the user.

##### Overview of the Network Simulation Tool

To set up a new simulation, the user creates a new Network Simulation Descriptor (NSD). Each NSD contains a complete specification that directs the tool to synthesize and execute simulations. NSD objects are composed and edited using a dedicated editing GUI. In particular, an NSD contains:

- A reference to a PLAN, and all its sub-objects (as shown in Figure 2),
- A simulation model for the environment that is used to generate sensor measurements and other “environment” events at simulation time,
- The sequence of result analysis and visualization operations to be performed on simulation output,
- Simulation-related parameters (length of simulation period, statistics collection granularity, HiL configuration *etc.*).

Once created, an NSD document can be used to execute new simulations at any time, by a “one-click” operation via the WebTop GUI. When a new simulation is launched on a given NSD, the tool performs the following steps:

1. The PLAN object is imported and information is extracted. This information comprises of WSN topology (location and type of nodes), the Connectivity Matrix, and the components and parameters defining each node type (NODEDEF and ROOTDEF objects). This information, together with other parts of the NSD, is compiled into a Platform Independent Model (PIM).
2. The PIM is validated, checking its consistency. If any inconsistencies are found, a detailed report is generated for the user and the simulation is aborted. Inconsistencies at this level are probably due to some user error (e.g., some NODEDEF has missing information). Also, the validation may create a number of warnings, which alert the user that something seems strange (e.g., routing seems misconfigured), but warnings alone do not abort the simulation.
3. The PIM is transformed to a Platform Specific Model (PSM). The PSM contains objects which describe the OMNeT++/Castalia simulation to be created. The transformation utilizes a library of Castalia-specific simulation models for the hardware and software components that define each node type (e.g., sensor types, radio chips, MAC and routing protocols, power consumption models, *etc.*).
4. The PSM is validated and if it is found to be inconsistent, a detailed report is generated and the process is aborted. A failure at this point is probably because of some problem in the tool (e.g., something wrong in the library of simulation models).
5. Based on the PSM, OMNeT++/Castalia code is generated, and compiled. Then, the simulation is executed by a suitable runtime manager.
6. The data output by the simulation is post-processed (including user-defined statistical processing) and a number of plots are created. These are sent to the WebTop GUI, in order to be presented to the user and the simulation terminates.

##### Supporting Environment Simulation with VectorL

In order to allow the user to specify simulation models for the environment, while hiding the underlying Castalia-based implementation, we designed a simple but expressive domain-specific language, VectorL. A more extensive discussion of VectorL appears in [46]. In a nutshell, there are three salient features of VectorL:

- Environment simulation models are typically models of physical systems. In order to code such models concisely, VectorL syntax looks very similar to vector calculus. Variables in a VectorL program are multidimensional arrays of reals, integers or booleans (scalar variables are semantically equivalent to 0-dimensional arrays). Complex values and strings are not currently supported, but may be supported in the future. In order to avoid looping constructs, which result in error-prone code, all VectorL expression operators are vectorized, similar to languages such as MatLab, R, yorick or Python/Numpy.
- At a higher level, VectorL programs are formed as collections of Event-Condition-Action (ECA) rules. Actions are sequences of statements. *Assignment statements* update the execution state, *emit statements* dispatch new events at a future simulation time and *conditional statements* implement conditional flow. When an event is dispatched, all actions for this event are executed.
- A program comprises of a collection of *modules*, allowing code reuse and testing. For example, in an air-quality simulation concerning three pollutants, the simulation model for each pollutant can be coded in a separate module, and the overall environment simulation includes all three modules.

**Figure 13:**
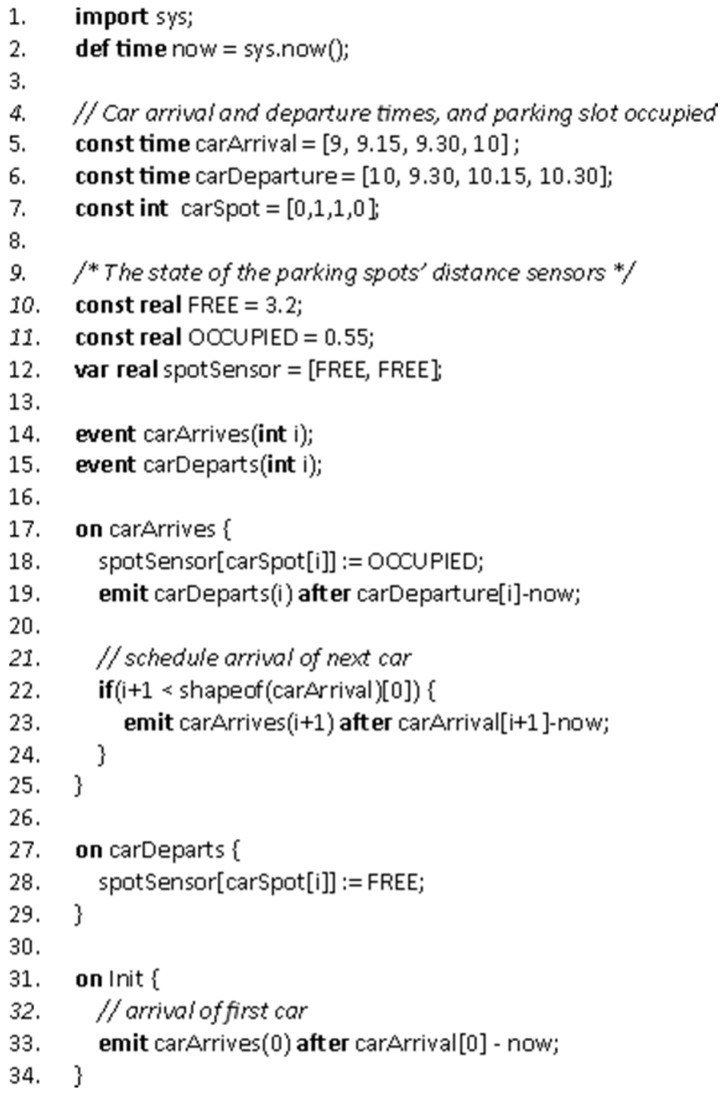
A simple VectorL program.

A simple VectorL simulation model is shown in Figure 13, which models a small, 2-spot parking lot, with four cars arriving. Three arrays (lines 5–7) hold the data per car, *i.e.*, arrival and departure times and parking spot occupied per car—in a more practical model, these data would be generated on the fly by random variables. It is assumed that there is a distance sensor located at each parking spot, measuring a distance of 3.2 m when the spot is free, and a distance of 0.55 m when the spot is occupied by a car. The current state for each parking spot sensor is maintained in an array (line 12). For each car, two events are emitted; a **carArrives** event, handled in lines 17–25, and a **carDeparts** event, handled in lines 27–29.

During a simulation, when a mote located at a parking spot reads its distance sensor, the value stored at the corresponding position of array **spotSensor** is returned.

Overall, environment simulation models are *much* easier to implement and test in VectorL than in C++. A web-based graphical integrated development environment (IDE) is used for VectorL code entry and editing. In this IDE, VectorL programs can be tested and debugged. VectorL programs are included into NSDs and are compiled into C++ when a simulation is generated.

##### Post-Processing Simulation Output

The network simulation tool provides a powerful yet intuitive method to determine the post-processing of events, based on a relational database. The output results from a simulation are collected into base tables. The user can declare derived tables (equivalent to relational views) by applying filtering, join, union and aggregation. Various types of graphical views can also be defined on tables (base, as well as derived). Supported graphical views include all kinds of traditional plots (histograms, line graphs, *etc.*), as well as displaying on the WebTop map view of the simulated network. The users do not need to be familiar with relational databases, or to write SQL code to define derived tables and graphical views; this is done in the NSD editor using a suitable GUI.

##### Hardware-in-the-Loop Mode

The network simulation tool is typically accessed as a web service, fully integrated with the rest of the WSN-DPCM platform. It is also possible to run the tool as a standalone program on a user’s workstation or laptop. Standalone execution is useful for simulations that involve hardware in the loop, which is connected to the local machine. When HiL is required in a simulation, the NSD must be edited to specify the simulated network location which is simulated using the hardware. HiL functionality is orthogonal to all other aspects of the simulation.

#### 4.2.2. Hardware-in-the-Loop Simulation

One approach to hardware-in-the-loop simulation is to use input signals coming from the simulation to drive the embedded system under test, thus extracting the output signals from the real setting. Through HiL developed extensions, the simulation environment (*i.e.*, OMNeT++/ Castalia) interacts with the real WSN environment (hardware as well as communication channel) to accurately incorporate in the simulation process some aspects of the wireless communication that are difficult (or even impossible) to model mathematically.

In the context of the project, we focused on two notoriously challenging to simulate aspects of WSN, namely the wireless transmission medium quality and the processing delay introduced by the hardware. HiL testing tools for WSN are anticipated to consist of dozens of physical sensor nodes.

**Figure 14:**
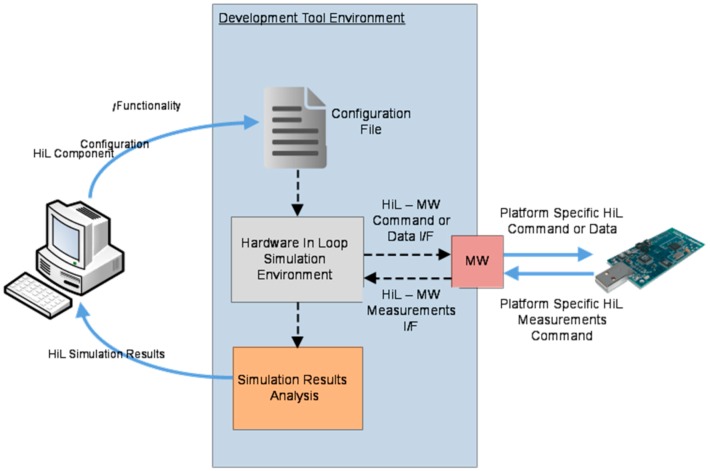
Interaction flow between the HiL Component and the hardware node.

For this purpose, we extended the OMNeT++/Castalia framework to support the interaction with real WSN nodes and support HiL components by issuing adequate commands towards the hardware as well as receiving respective feedback regarding the performance/behavior of the hardware, which is then integrated into the simulation process. Using the WSN-DPCM network simulation GUI, the user is able to select whether the plugged-in real sensors are to be considered (effectively activating the HiL features) or not. In this context, the developed HiL Simulation Component is able to connect with the hardware nodes running a modified version of the application firmware that includes the interface for data and command exchange with the simulator at simulation run-time. More specifically, the HiL component issues commands and data which are to be conveyed to the actual mote through DT-MW functions. These commands will effectively instruct the hardware to perform well defined and beforehand known operations and collect the respective measurements. The information on operation execution and on respective measurements are then conveyed back to the HiL component, which incorporates them in the simulation process. An abstract view of the interaction flow of the HiL component with the real hardware is shown in Figure 14.

We use of the “system()” function to implement the forward channel, which can execute a command with optional command-line arguments in a shell on a UNIX-like operating system. We executed the Java program responsible for the communication with the motes, along with the required arguments. The reverse channel is implemented using a named pipe.

**Figure 15:**
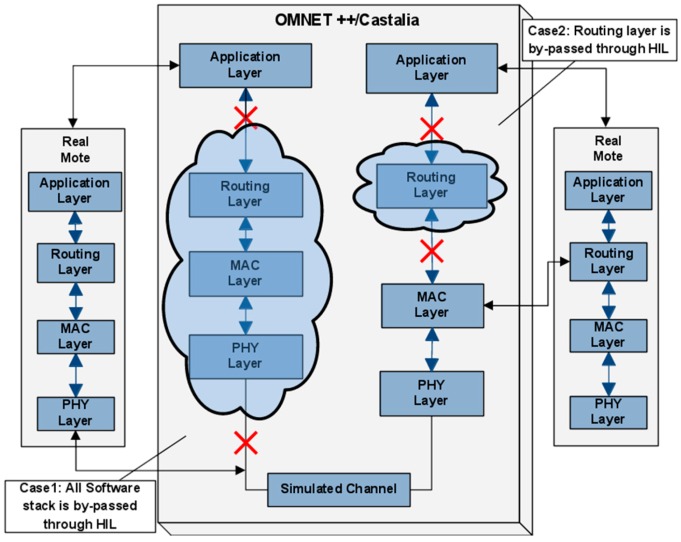
Processing Delay HiL Scenario.

As a proof of concept, we have considered two HiL Simulation Components which tackle aspects notoriously challenging to model in a WSN simulation. The first aspect concerns the hardware processing delay, which in most simulation environments is omitted in the overall delay evaluation. In that respect, for each WSN node the user is able to define which parts of the software stack will be simulated and which will be executed by the available hardware. Therefore, based on specific codes issued to the hardware, the user effectively selects one or more modules of the software stack of a node, to be simulated or executed on the real hardware. In Figure 15, a possible configuration is presented.

**Figure 16:**
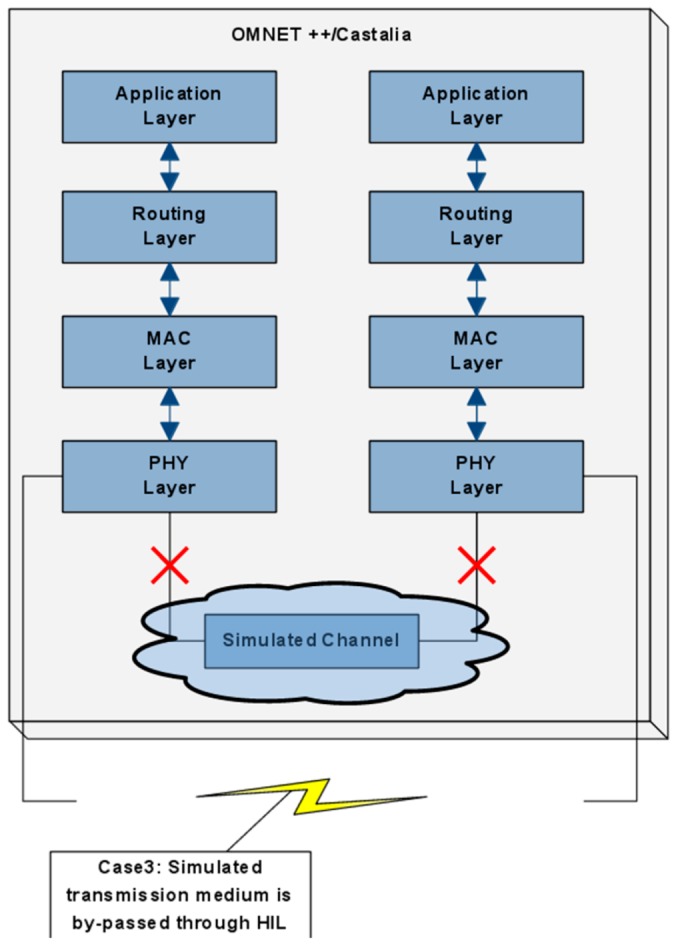
Wireless Medium HiL Scenario.

In this case, the user has chosen the routing and the MAC layer of a virtual node to be executed by a physical node, instead of being simulated, while for the second node, the user has chosen only the routing layer. The second approach aims to accurately capture and integrate in the simulation environment the dynamics and unpredictable characteristics of the wireless channel. The above scenario is schematically shown in Figure 16, which allows one to accurately measure the real RSSI and BER, and thus to take into consideration an objective notion of communication link quality.

#### 4.2.3. Middleware

**Figure 17:**
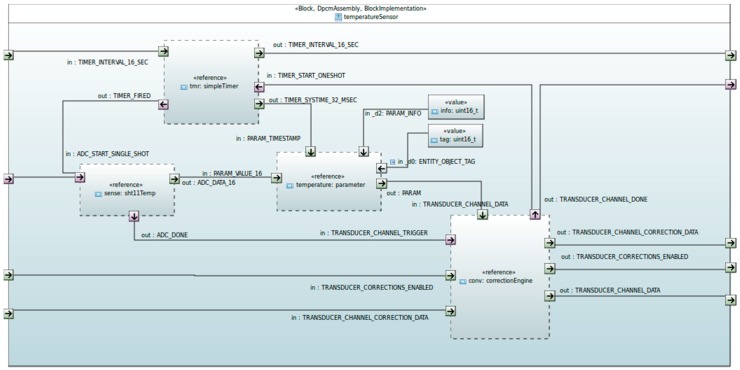
Middleware sensor assembly example.

The WSN-DPCM Middleware is responsible for the definition of the common abstractions and the realization of their interfaces for components of functionality typically existing in WSN-based systems. The main objective is to enable high level compositions over heterogeneous embedded programming platforms, promoting portability and reconfigurability, as well as interoperability based on mappings to international standards and industry driven specifications. It defines a core component execution framework which includes design-time configurable inter-component communications, execution scheduling and resource monitoring mechanisms. Based on this and on common interfaces, it provides implementations of components for HAL, Network and Platform layers, hiding the lower-level details (hardware and OS drivers), implementing the available networking and distribution mechanisms, and offering interoperable services to local or network applications. Middleware component abstractions are provided as UML/SysML block descriptions of leaf Function Blocks (executable code) and Assemblies (connectable compositions of other blocks/assemblies with input and output ports, as shown in Figure 17). A component developer, who typically is an expert programmer, has to implement the interfaces of the component to the configuration and runtime sub-systems, and provide SysML block descriptions with all attributes required by the defined WSN-DPCM UML Profile [47].

Every assembly (its .uml model) can be fed in the developed Xmi2x WSN-DPCM parser, which generates the instantiations, links and configuration files ready to be inspected or manually added to a previously generated device source code tree, or ready to be compiled alone as a relocatable generic binary that can be dynamically loaded into a running device.

The middleware run-time environment provides C and NesC bindings of the various block interfaces and implementations, as they also appear in the toolset repository, based on the corresponding Block, Parameter and Variant argument models of IEEE 2145-1, but with identification tags mapped restricted in size and semantics, in order to comply with the OMA LWM2M addressing scheme (type ID/object ID/parameter ID). It defines its own build environment, which combines and abstracts the underlying operating environments (Contiki [48], TinyOS [49], ChibiOS [50] and RIOT [51]). This combined environment along with the captured booting process also allows one to selectively combine the interleaved execution of two run-to-completion schedulers, either over bare hardware or inside a RIOT or ChibiOS thread, thus allowing the coexistence of blocks with internal implementations in either OS.

**Figure 18:**
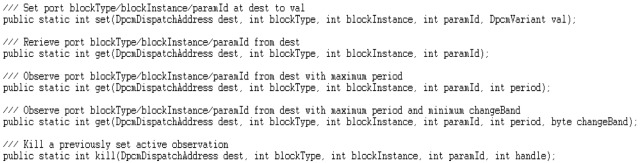
High level MW component remote management API (Hand-held java library).

In order to simplify the remote access to various block parameters (ports), the middleware provides also high level implementations of Java APIs in order to be used by Java-enabled gateways and configuration and monitoring clients such as the CMT Hand-held, abstracting all the MW functions as set/get accesses to a selected block instance or port, following the OMA addressing scheme, as shown in Figure 18. The Java APIs offer direct network access either to the WSN, through an additional C/NesC programmable embedded interface, or to the web side of a gateway.

### 4.3. Tool for Network Commissioning and Maintenance

The Commissioning & Maintenance Tool (CMT) [52,53,54] plays a significant role in overseeing deployed WSN networks and providing the WSN-DPCM platform with network feedback information in real-time. The data collected enable online inspection and offline analysis procedures aiming to both refine network setup and to validate the overall deployment assumptions and design decisions from the operational and behavioral points of view.

The CMT is mainly devoted to the operation of WSN-based networks, but it equally offers a valuable support for network deployers, testers and maintainers during installation, setup and commissioning operations of a deployment. More in depth, CMT assists the developer to prepare the nodes for field deployment, during the actual field deployment operations, and for node operation monitoring and debugging after deployment, for long-term maintenance. It uses the programming images and configurations generated by the Development Tool for network node types and profiles, and the deployment model provided by the Planning Tool. Once the WSN is deployed in the field and activated, the actual operating conditions of the nodes are collected by the CMT and can be compared with the results of the RF and network simulations to correct and optimize various network parameters.

The CMT is integrated with other platform systems through RESTful web services. That is also the case for the integration with the middleware, which provides run-time access to network data including network traffic, sensor measurements and nodes status at various levels. Furthermore, the communication with the Project Repository is also based on a RESTful API, which provides access to all deployment-related data models and simulation parameters that are considered within the development and planning phases by the Planning Tool and the Development Tool. Thus, the communication and synchronization with the Platform relies on concrete data model items, used as input, which are retrieved from the Project Repository on session start-up and during its regular operation. Given its nature, the CMT systems need to obtain the high-level design specifications of the Project and the network Plan for the deployment to be assisted. Through model entities, the CMT systems are able to configure internally and gain insight about the composition of the network in terms of number and types of nodes comprised, their spatial distribution, connectivity, communication protocol attributes, supported data categories, parameters and services, among many others.

On the other hand, the CMT keeps relevant data collected in database historical registers over time for offline inspection and analysis. However, it is also able to produce valuable output in the shape of visual graphs and online reports, which are automatically uploaded to the Project Repository as essential feedback elements to drive potential refinements/corrections of the deployment plans, network designs and even node firmware adaptations. As described in the following sections, the Tool is heterogeneous in nature and comprises a variety of software systems:

- A hand-held device (CMT-HH) for network deployment and in-field monitoring; it provides an on-site GUI accessible to application domain experts (tablet-like touch screen device).
- A client-side web application that provides an off-site GUI for engineers to perform long-term monitoring, control and easy inspection of WSN network from a standard web browser.
- Server-side services that enable the communication with the WSN-DPCM platform systems and modules, and other mission-critical functions (data analysis and housekeeping, consistency checks, among others).

The main capabilities and functional objectives of the CMT within the WSN-DPCM Platform are:

- WSN real-time data and metrics monitoring.
- Off-line analysis and statistics over collected network data.
- UI data visualization (graphical & textual).
- WSN nodes and control through network commands.
- Software-aided support for WSN deployment validation.
- Support for in-field WSN commissioning and maintenance.

Besides the monitoring and network control functions offered by the tool, text presentation and visualization services represent very useful methods for CMT operators to figure out, commission and troubleshoot undesired behaviors, such as incorrect network operation/behavior due to data inconsistencies, communication problems or erroneous device setups and configurations during network deployment and regular operation.

#### 4.3.1. Hand-Held Device for Network Deployment and in-Field Monitoring

During the development cycle of a WSN-based application, in-field configuration and performance assessment of wireless sensor nodes by means of integrating well-defined commissioning and debugging techniques during deployment can significantly reduce the verification and final release of the system. Moreover, for platform developers this stage represents a key validation and/or refinement opportunity for the previous development phases regarding modeling, simulation and code generation processes. This is the main reason why a new on-site commissioning mechanism is proposed and integrated in the WSN-DPCM toolset. It supports a comprehensive deployment methodology for network/node parametrization, installation and configuration, as well as in-field runtime behavioral analysis on the actual hardware and software elements that compose the sensor devices to be deployed. In order to carry out this approach, a CMT Hand-held device (CMT-HH) [52,53,54] has been implemented as part of the toolset, which integrates several heterogeneous technologies into a unique HW-SW platform. The main target of the CMT-HH is to provide deployers with expert/non-expert capabilities that help them accomplish the deployment activities in an efficient, optimized and automated way.

**Figure 19:**
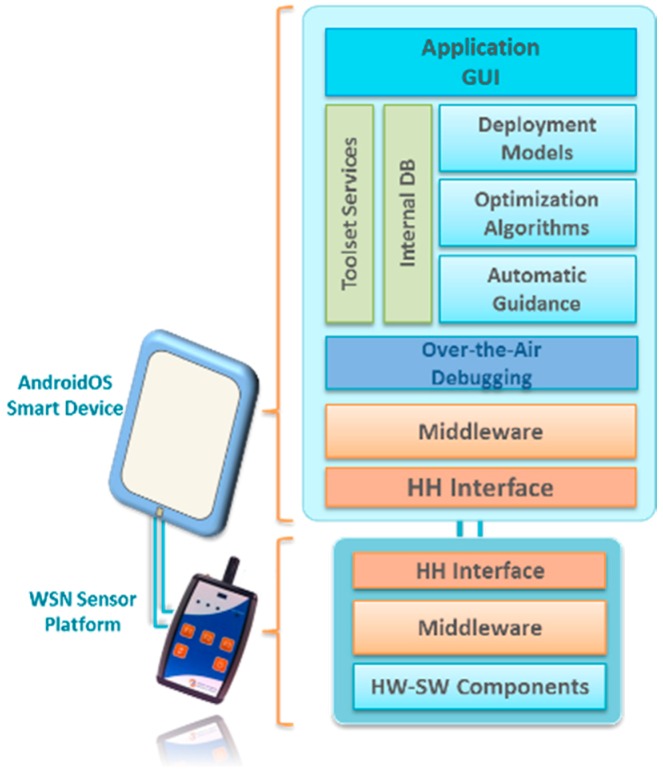
Overall HW-SW structure of the Hand-held device for the Commissioning and Maintenance Tool.

The system architecture of the CMT-HH is composed of two main elements upon which the deployment tool technologies are integrated. On the one hand, a smart device that supports the Android operating system is considered as the mobile component to perform the on-site deployment and commissioning activities. On the other hand, the sensor node platform is integrated with the aforementioned device by using a common interface connection between both components in the form of a USB-host mode configuration, in order to provide the CMT-HH with the wireless communication interface to the sensor network deployment (such as the IEEE 802.15.4-based connection), as shown in Figure 19. This low-level communication interface serves as the over-the-air debugging channel to carry out the in-field node/network configuration, monitoring and analysis tasks.

Based on this approach, the CMT-HH includes the HW-SW components shown in Figure 19, in which the main elements of the proposed platform are highlighted. First of all, the CMT-HH integration with the WSN-DPCM toolset follows a model-based approach, by means of using the common Project Repository via a web service interface scheme. The CMT-HH retrieves two main structured models as input to determine the in-field deployment strategies as well as the system preparation, which are represented as JSON objects.

One is the network/application model, where the functional blocks in the node are defined (sensor and actuator elements, properties and configurable parameters, hardware device features, among others) as well as network information regarding node positioning, communication parameters and node type for topology definition. This model is the result of planning and development stages of the application development. An object-oriented approach is followed to configure the internal deployment model of the tool from the basis of the aforementioned network model retrieving. Sensor node definition is established by functional block instantiation and parameters that outline the node platform implementation (such as, for instance, sensor and property configurations that can be accessed and modified at runtime). As explained in following paragraphs, this component instantiation follows an object/parameter addressing scheme to establish a coherent block interface with the middleware-based node embedded components.

**Figure 20:**
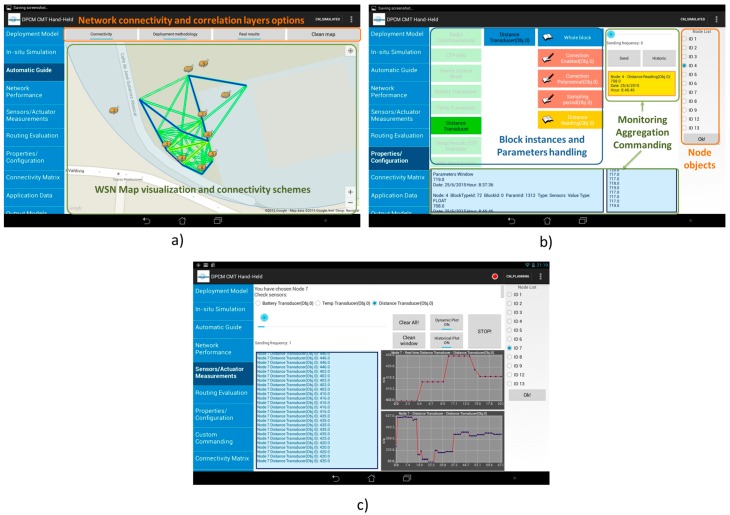
General view of Hand-held top-level interface in Commissioning and Maintenance Tool. (**a**) Network connectivity and node correlation scheme layers; (**b**) Runtime node functional components management; (**c**) Data aggregation and on-site monitoring/commanding.

The other is the radio connectivity model, which estimates the connectivity between sensor nodes so that strategies for improving the communication coverage can be applied accordingly (in combination with node placement information and configuration properties). This connectivity model is generated through simulation during the planning stage of the toolset. The basic information that is used to build the network connectivity scheme is on the one hand the bidirectional link metrics between pair-points (particularly considering RSSI and LQI parameters), and, on the other hand, the radio communication configurations such as TX power modes and wireless channel assessment. This connectivity scheme is subsequently analyzed and compared with the actual performance of the sensor nodes under deployment/maintenance, by means of generating a real connectivity scheme in accordance with the runtime node configuration process. As shown in Figure 20a, both simulation and real connectivity maps can be overlapped so that deployers can study the influence of on-site factors and/or installation modifications on the overall performance of the wireless sensor deployment, in addition to deployment methodology sequence outcome. This representation allows the provision of parameters regarding isolated nodes, redundant communication areas and critical points of the network.

Four main elements can be distinguished within the system implementation of the CMT-HH, which also define the system functional flow once the internal data management system is supplied with the aforementioned input models.

First, an in-field deployment methodology built upon run-time optimization algorithms, so that the users are provided with the most effective configuration and installation routine for the wireless sensor nodes to be deployed in the target scenario. This optimization engine focuses on identifying on the one hand the best deployment sequence to assure the cohesion of the network with an optimized power and resource consumption during the configuration routine; and, on the other hand, to find key-strategy-points of the WSN with which the network topology scheme is achieved with a minimum control packet dissemination cost.

Second, an on-site comparative analysis of the actual wireless communication performance of the sensor network with the theoretical simulation models generated at planning stage. Apart from the network/node link correlation schemes based on connectivity metrics and radio module parameter reconfiguration to improve the overall networking map, an experimental evaluation of multi-hop communication mechanisms can also be realized based on routing protocol information provided by the sensor platform during deployment considering the monitoring and computation of metrics such as packet loss rate and retransmission counts, as well as routing metrics for path evaluation.

Third, run-time functional sensor node reconfigurations that allow to modify specific properties to improve wireless network performance, especially to reduce power consumption (such as the sensor sampling rate and transmission period, sleep mode configurations, transmission power) and network/application debugging tasks. This capability is supported by the Java-based middleware interface functions to access the implemented embedded components of the sensor nodes from the CMT-HH tool, in accordance with the input network model definition. Considering the node objet, block instantiation and parameter identification, a particular functional component of the target sensor node can be addressed and configured at runtime, as shown in Figure 20b, where different block instances and node component parameters can be accessed, updated and monitored. This abstraction layer has been integrated as a two-side interface between the upper debugging and evaluation layers and the USB-host hardware controller of the mobile device.

Fourth, automatic and dynamic generation of the output models related to system performance assessment and analysis/optimization process carried out during deployment activities, to provide a comprehensive feedback for the toolset based on data gathered in the real environment scenario.

After field deployment, CMT-HH provides output models associated with the underlying information obtained during in-field testing, debugging and optimization process and whose reports can be used to enhance the simulation models and planning strategies. Furthermore, deployers are provided with direct data processing visualization entities as shown in Figure 20c, where runtime WSN monitoring and statistical data aggregation is produced based on the over-the-air connection of the on-site tool and the sensor nodes under commissioning.

#### 4.3.2. Application Server and Network Long-Term Monitoring and Maintenance Tool

Accompanying and complementing in-field deployment activities, an off-site application for network commissioners and network maintainers was developed to simplify remote, long-term commissioning of operative networks.

Continuous monitoring and visibility over network data and events are key aspects taken into account by the CMT server modules and applications [52,53,54]. Together with the ability to change node behavior through remote partial reconfiguration, the ability to analyze network throughput are essential features for the validation process of a deployment and, even more, for corrective and preventive maintenance of long-lasting WSN networks. 

CMT server modules retrieve from Project Repository different models generated in various phases of design and planning for deployment of the network and use them for automatic self-configuration. This capability allows a perfect alignment of the virtual and the physical worlds in a WSN deployment.

**Figure 21:**
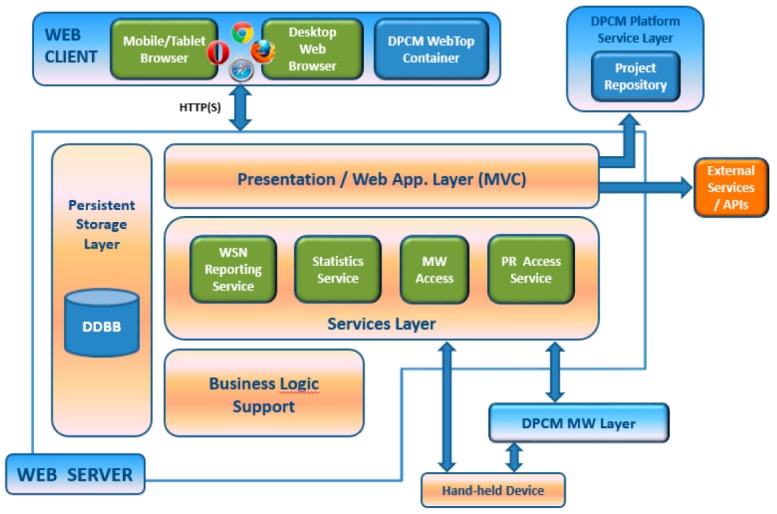
Overall software architecture and interfaces of the Commission and Maintenance Tool server component.

The support offered by the CMT Server comprises a variety of modules that are integrated with and/or make use of key WSN-DPCM platform services and systems. Figure 21 shows an architectural view of the CMT Tool and its interfaces.

**Figure 22:**
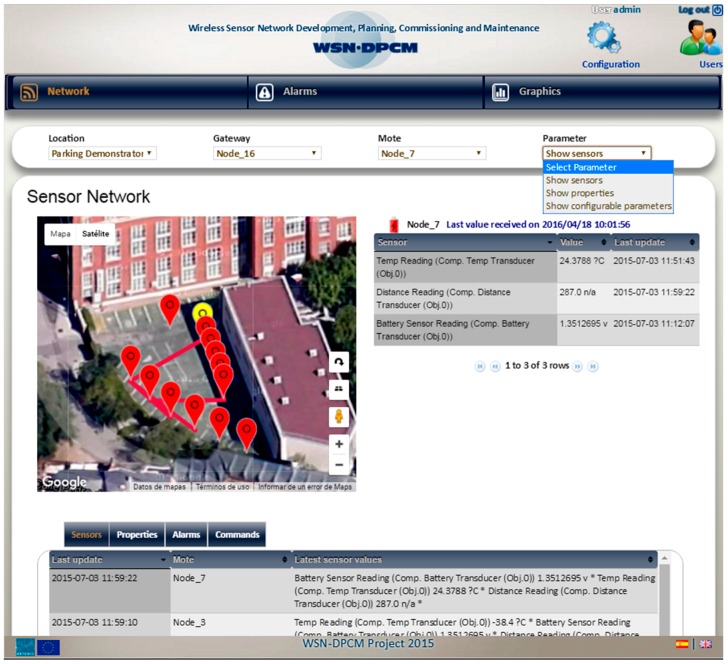
General view of the top-level interface of the Commissioning and Maintenance Tool Web Application.

At application-user level, the CMT Server suite provides an administrative web application UI (Figure 22), accessible from the Toolset run-time environment, which allows to remotely monitor and control the operation of the network for long-term analysis and maintenance. This web-accessible tool does not require specific expertise to be used by the people in charge with network deployment, while also providing the expert users with extended information on the network. Among the most important features provided are:

- Data formatting and visualization procedures: Data from Sensors, Network Diagnostics (routing, RSSI/LQI), Node Diagnostics (battery level, status of operation), Communication metrics (packets TX/RX/dropped, congested links), Hand-held reported information.
- Support for remote reconfiguration of network nodes.
- Event/data logging and online data charts generation. Data reports export options.
- Configurable baseline of data inspection rules, associated to alarms and notifications.

At a service level, the CMT Server enables a set of services to access the WSN-DPCM Platform Project Repository on the cloud (data models proxy service) and to collect real-time network telemetry data (sensor measurements, network status, node status parameters *etc.*) The interaction model with the WSN-DPCM platform follows a model-driven approach, founded in web service communication APIs. These interactions include:

- Model files retrieval from cloud-based Project Repository.
- Information exchanges with the Middleware run-time layer (data parameters, ACKs, data/command requests), data consistency checks and storage.
- Collection and storage of deployment reports from CMT hand-held (HH) devices.
- Proxy service for the HH devices (digested network model download).

To sum up, all CMT support services and in-field/off-site solutions combined represent a reliable set of software-based utilities provided by the WSN-DPCM toolset to assist network deployers and maintainers in the tedious task of installing, setting up, validating and optimizing a running WSN network, and to reduce the number of iterations with respect to the traditional trial-and-error approach.

## 5. Use Cases

The usefulness of the WSN-DPCM Toolset was evaluated within the project through creation, planning, simulation and deployment of two WSN-based demonstrator prototypes.

An Outdoor Parking Demonstrator [2,3] was implemented as a WSN installed in a parking lot which is able to detect free parking slots and to guide the drivers in real time to them.

An Air Quality Demonstrator [4,5] was realized by deploying a WSN near an industrial site close to a city to monitor in real-time atmospheric concentration of several dangerous gasses or particles: LPG, Natural Gas, Carbon Monoxide, Coal Gas, Liquefied Gas and Dust.

### 5.1. Assisted Parking

This outdoor demonstrator [37,55] aims to provide a smart parking implementation in which the users can reserve, manage and monitor the state of the available car slots in the application scenario, so that a more efficient control of the parking space is carried out by using a wireless sensor network created through the proposed WSN-DPCM toolset.

**Figure 23:**
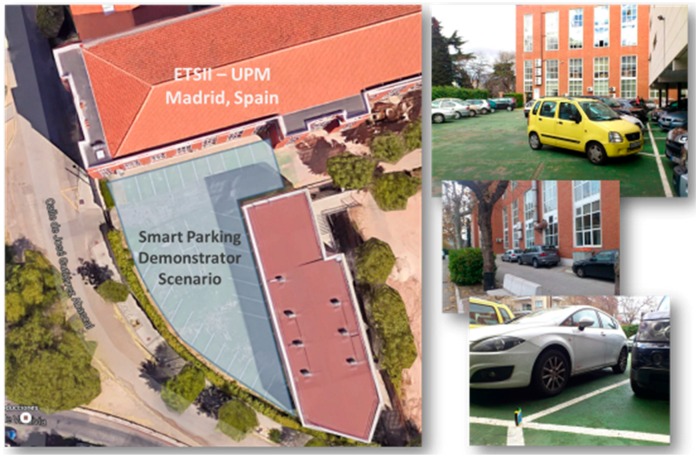
Application scenario for the smart parking demonstrator.

The target scenario for the deployment of the WSN-based demonstrator was a car park space of the Escuela Técnica Superior de Ingenieros Industriales (ETSII) as shown in Figure 23, which is an engineering school that belongs to the Universidad Politécnica de Madrid (UPM), in Spain.

**Figure 24:**
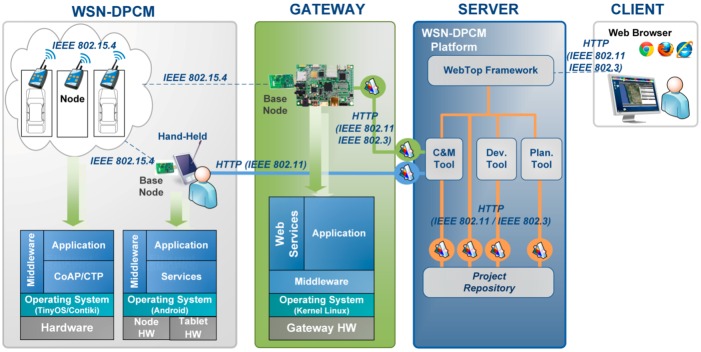
WSN infrastructure for the Smart parking demonstrator scenario.

The overall architecture of the proposed WSN for monitoring and controlling the smart parking demonstrator is shown in Figure 24.

The prototype comprised a WSN with 14 nodes and one root device. The hardware elements that composed the deployment are based on the following items:

- Sensor nodes: the Inetsis platform was used as the sensor devices for measuring and processing the occupancy of the car slots in the parking scenario. They have a TelosB-like design including IEEE 802.15.4 radio communication modules and a low-power MCU for local processing.
- Gateway device: it includes the low-rate communication protocol for gathering run-time information from the WSN and provides a remote connection to the distributed sensors by using Ethernet/Wi-Fi communication. An embedded PC was installed with a sensor node connected as the root of the network.
- Server: the server side refers to a distributed array of different machines that provide the WSN-DPCM support services as well as the remote commissioning capabilities of the system. In addition to these, the top-level user application services are also provided regarding parking reservation and monitoring capabilities.
- CMT-HH: The Hand-held device was used to carry out the deployment, configuration and in-field performance evaluation of the sensor network at the target area.

Regarding software implementation, several elements can be distinguished within the developed prototype as outputs of the WSN-DPCM toolset application development.

First, the embedded software that runs on the sensor platforms was obtained from the integration of the middleware functional blocks, as a result of code generation during the development stage of the system.

Secondly, the capabilities of the CMT-HH were used to analyze and optimize the in-field deployment of the sensor network considering the generated planning and simulation models for this particular scenario, in addition to providing run-time on-site verification of sensor implementation and communication capabilities. CMT-HH, Gateway and CMT Server sides included the corresponding middleware interfaces to properly interact with the distributed nodes in real time. 

**Figure 25:**
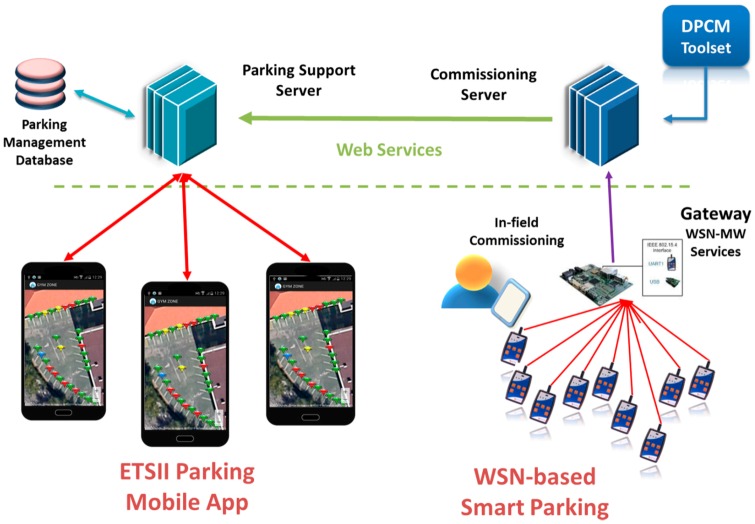
Underlying structure of the smart parking user application.

Last but not least, in order to provide a user-level smart parking application, additional elements were implemented to emphasize the usability of the demonstrator from the user perspective. In this context, the car parking space was controlled using a designed Android application for Smartphones, in which the users can reserve specific slots as well as to monitor the occupancy state and availability of the places in real-time. As shown in Figure 25, such capabilities were supported using a communication infrastructure composed of a parking management server that retrieved information from the CMT Server, and then provided web-based services to the users of the parking application. 

**Figure 26:**
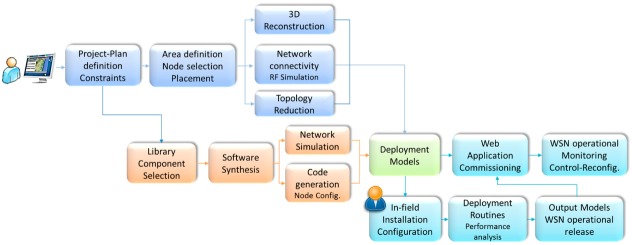
Overall end-to-end flow of the demonstrator implementation by using the DPCM toolset.

The design of the service network for this real-world application scenario was entirely done using the WSN-DPCM Platform’s services and tools. The creation, deployment and validation of the demonstrator is carried out following the end-to-end scenario flow shown in Figure 26, in which the proposed toolset capabilities are comprehensively applied to obtain the target WSN application, considering an iterative process for system refinement and implementation enhancement through simulation and testing procedures.

In the first phase is created the project entity which encompasses the application objectives and constraints to start the planning and development of the WSN. The input information required also includes the target area to be covered by the smart parking, as well as the surrounding buildings for a more realistic RF simulation of the application environment. For this, the 3D reconstruction engine is applied to automatically generate the building information. Moreover, the type of parameters to be measured is defined, considering the sensors for vehicle detection such as ultrasonic or infrared light intensity.

The second phase comprises the creation and management of the network plan, upon which nodes equipped with sensor detectors will be located in the parking slots of the target area, also defining the gateway placement information. Based on this, simulations are triggered in order to improve node positioning and configuration settings, taking into account RF and network connectivity simulation as well as topology reduction.

During the third phase, the network simulation synthesis and software synthesis are performed alongside the HiL simulation to collect information related to communication and measurement delay for car detection, in addition to sensing and radio coverage evaluation. Then, the application code is generated by synthesis in accordance with the outcomes of the aforementioned process, so that the corresponding node firmware can be produced.

The field deployment, installation and configuration tasks represent the next phase which is supplied with the network models and functional components that compose the overall system implementation, based on the outcomes of the previous stages. The deployment model encompasses the placement and installation of 14 sensor nodes plus the inclusion of two more nodes for routing purposes as a result of the simulation optimization stage. Then, based on the location coordinates, network connectivity scheme and deployment methodology, the installation and configuration process is assisted by means of the CMT-HH, while the operational release is finally verified by the CMT web application, which registers and presents the output models of the in-field commissioning activities for further system optimization and feedback analysis.

As a summary of the application implementation of the smart parking demonstrator based on the DPCM toolset, during the planning phase RF connectivity and topology-related services were extensively used and decisively contributed to the successful deployment carried out in later stages of the demonstrator-building process. 

Likewise, all deployed nodes were equipped with MW modules which simplified the setup activities by providing remote reconfiguration capabilities. Furthermore, the embedded MW support proved particularly useful in the adaption of nodes configuration and their behavior during deployment and maintenance phases. 

The Network Simulator also played an important role in inspecting the functional profile of the design, before allocating personnel and resources to in-field activities. The results of such simulations comprised communication and topology aspects and operative aspects of embedded software, and helped optimizing design decisions taken iteratively through design-and-simulate cycles. During the installation phase, CMT tools used the deployment models and artifacts produced to place nodes and configure them according to design specifications, as well as to analyze and verify the performance and operability of the whole network. 

Overall, the support and assistance provided by the WSN-DPCM tools and services helped reduce the number of iterations in design, thus accelerating and simplifying the transition from a virtual, simulated network to a real, fully-functional solution, compliant with demonstrator and user requirements.

### 5.2. Air Quality Monitoring

**Figure 27:**
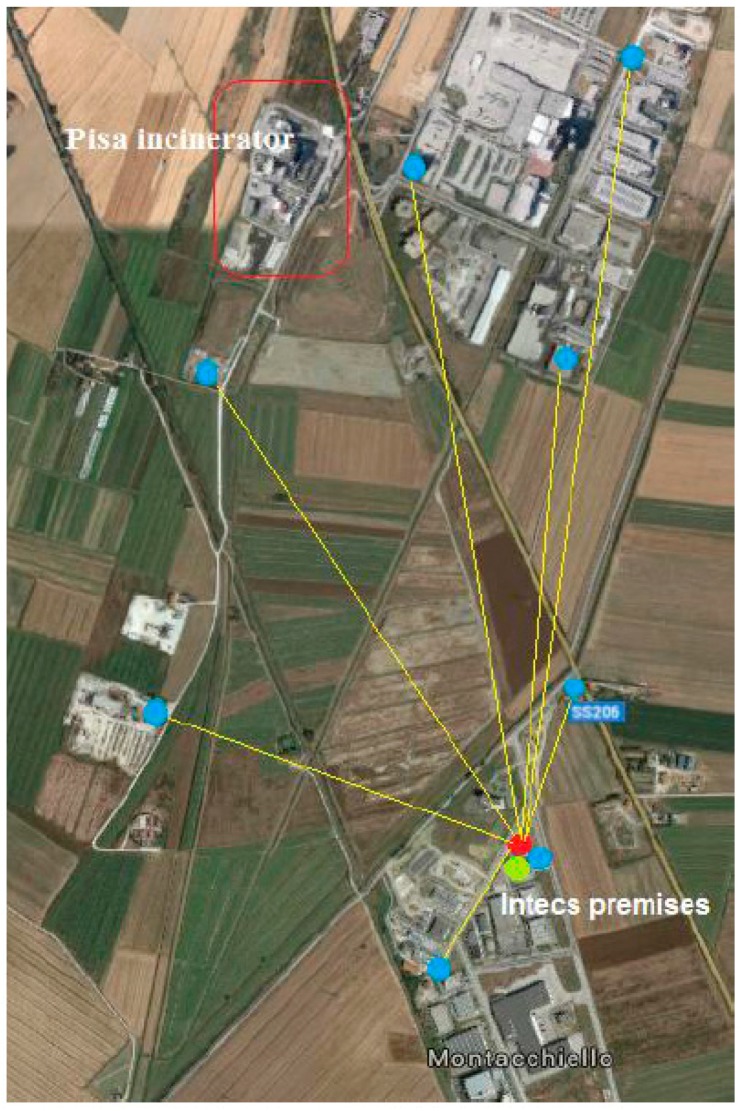
Application scenario for the air quality monitoring demonstrator.

The Air Quality Demonstrator [37,55,56] was deployed in the industrial zone of the city of Pisa (Italy) in agreement with the local authorities. The main goal was to collect concentration measurements on harmful gases such as Carbon Monoxide, Coal Gas, Liquefied Gas, LPG, Natural Gas, Town Gas. The sensors were installed in the area to monitor gases concentration (see Figure 27).

WSN data acquisition for environmental monitoring is challenging, especially for open natural fields. These usually require high reliability and long maintenance-free operation while the nodes can be exposed to variable and extreme climatic conditions.

**Figure 28:**
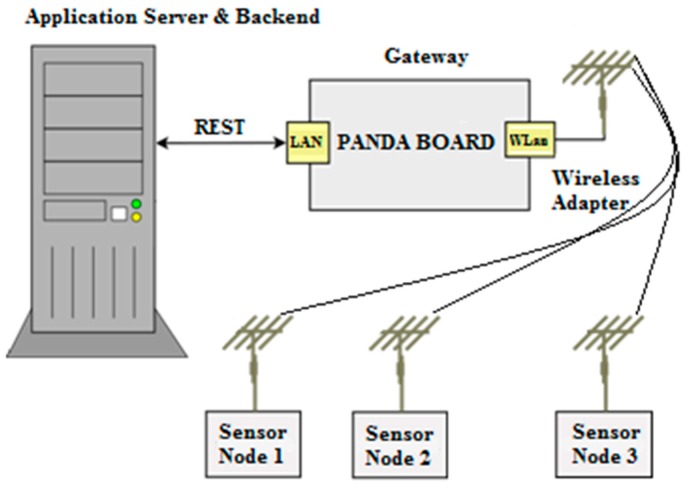
Main components of the network.

The main network components are one server to save data and provide end user services; one gateway to collect field data; and eight sensors to measure air quality (sensor nodes, see Figure 28).

The main purpose of the server is to receive, store, and provide access to field data. It bridges the low power communication segments with latency-energy trade-offs, and the fast and ubiquitous end-user field data access. It provides interfaces for reading sensor nodes values. A central software engine controls the server operation and the access to the main database. It is written in Java and has low-level layers written in C and runs on a Linux operating system. The sensor nodes are connected to the central gateway using an XBee RF Modules interface.

The main role of the gateway node is to collect, process, and forward to server the field data received from the sensor nodes. The gateway software is composed of: a first component, used to manage high level interface like the REST interface and the relative JSON messages; a controller, that contains the core functions of the gateway (collect data, manage topology, send message, manage component interaction); a second component, used to manage the sensors interactions. The gateway hardware equipment is a Panda Board Rev A2.

First, the Planning Tool was used to simulate 13 deployment plans in order to optimize different aspects, especially node types and positions. In fact, using the PT RF simulation tool and its topology services (Connectivity Matrix, Topology Evaluation and Reduction) we discovered without any in-field tests that some nodes were isolated from the gateway either because they were obscured by buildings, or because they were deployed at an inadequate height or too far away from the gateway. Each deployment plan simulation, including RF simulation, connectivity matrix evaluation and topology analysis, required about 7 min (most of the time being spent for RF simulation) for this scenario with ten nodes and a linear size of about two km, by using a four core Intel(R) Xeon(R) CPU E5440 @ 2.83 GHz with 3 GB RAM. Accordingly, all this phase only required about two hours and a half, also including the time needed by the operator to provide input data and to read the simulation results.

At the end of this process, we obtained two deployment plan candidates for implementation and proceeded with the use of the Development Tool for node hardware-software synthesis and network simulation. Since we were interested to use ChibiOS real-time embedded operating system for this experiment, we modeled and added to the Development Tool library a suitable set of components for this OS. Moreover, several DT library components were designed to model the sensors that we have selected for this application mainly for hardware requirements, configuration parameters and interfaces. Additionally, to support middleware-like services, we have added to DT library components with blocks that were designed to communicate with the application server and allow run-time retrieving and setting of specific values for in-field node monitoring and configuration.

With these supporting elements, we used the Development Tool to explore several node variants and their network-level effects. For instance, by changing the sensing requirements in the specifications of the nodes we obtained different node software suitably configured for programming (e.g., with the node IDs from the planning) and also hardware specifications that make sure that the node is capable to support the operation and sensing functions at run-time in each of those configurations. For each of these, the DT generated also the models for network simulation. From these simulations we discovered that the packet transmission rate achieved with the selected schedule was not suitable. Thus, we iteratively refined the planning and the requirements until the network operation proved satisfactory.

In the end, the sensor node that we selected was composed of:

- A Nucleo Development Board, NUCLEO-F401RE, which enables to build and evaluate the prototype with the STM32 F4 family of high performance microcontrollers in an embedded application;
- An Xbee Zigbee Shield that allows an Arduino board to communicate wirelessly using Zigbee;
- A Digi International XBP24BZ7SIT-004, which is an embedded RF module that provides low-cost, low-power, wireless connectivity using the ZigBee PRO Feature;
- A MOSFET Power Controller to switch a battery supply on and off;
- A Grove-Gas Sensor (MQ9-MQ5) that is useful for gas leakage detecting since it can detect Carbon Monoxide, Coal Gas, Liquefied Gas, LPG, Natural Gas, Town Gas;
- A Grove-Temp & Humi Sensor, which is a temperature and humidity sensor that provides a pre-calibrated measurement.

**Figure 29:**
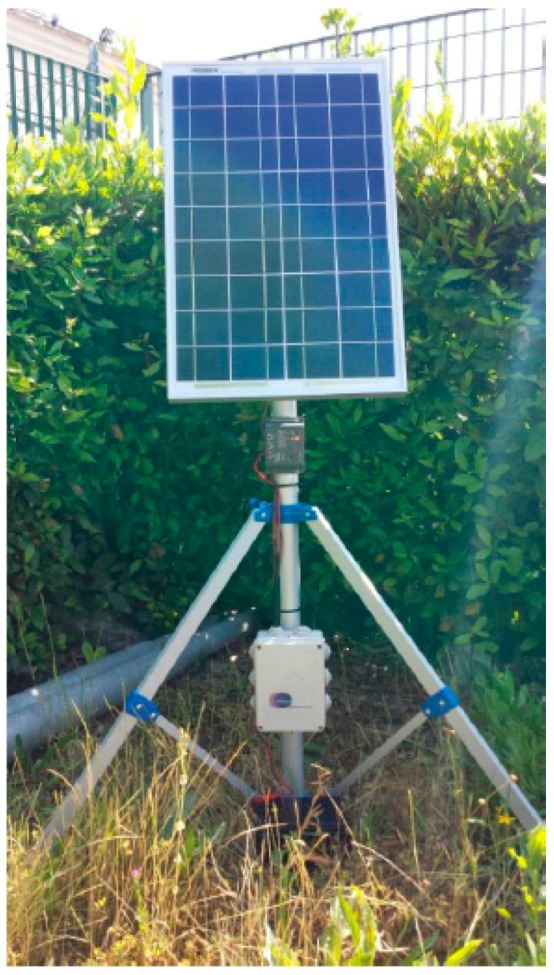
One of the nodes (enclosed in the white box) with its solar panel.

For proper operation, both node types need batteries, solar panels, a communication module, and antennas for in-field and long-range communications. Figure 29 shows one sensor, with its solar panel.

Network planning and application development took about two days of work. After this, the physical deployment of ten nodes in the field took two more days of work, in which two engineers went by car, sometimes in poor weather conditions and, for some nodes, on rough terrain. We can estimate that a trial-and-error approach, in which we would have to physically install more than ten configurations and topologies, would have probably required more than one week just for the physical deployment of the network, and an additional week to perform in-field tests and optimization. Thus, the use of the WSN-DPCM platform saved us significant time and costs.

Using the middleware-like services embedded in the node software by DT synthesis according to node specifications, the CMT was able to connect at run-time to node software. The CMT enabled us to perform monitoring operations directly from our desk, with no need of in-field tests. This way we were able to continuously monitor the operation of the WSN deployed in the field through CMT interfaces to check that our WSN was properly operating.

## 6. Conclusions

We presented the WSN-DPCM integrated toolset for WSN application planning, development, commissioning and maintenance, which has been specified, designed and implemented within a UE ARTEMIS-JU project. The toolset aids and assists the application-domain experts, may have limited WSN expertise, in the task of efficiently developing WSN applications from planning to lifetime maintenance. The main benefits of the WSN-DPCM toolset at platform level are:

- Reduction of trial-and-error WSN deployments, thanks to the extensive multi-level simulation support (RF simulation, network simulation).
- MDA-based automatic system synthesis and code generation, through reusability of software and hardware components from extensible libraries.
- Simplification of the design, deployment and maintenance phases of real-life deployments by using a software-assisted approach.
- Middleware support for abstraction from the complexity of WSN environments composed of a multitude of embedded platform technologies.

The benefits of the proposed toolset have been evaluated using two case-studies in two different WSN application domains: one that detects the occupancy state of parking places and one that detects the presence of concentration of potentially harmful agents in the atmosphere. These applications have been chosen to exercise the toolset for the design of typical WSN application elements, such as different network topologies (cooperative short-range mesh networking for parking and star-based long-range for air quality), for design space exploration to instrument the nodes with the proper types of sensors and optimize their operation, and for optimal field deployment simulation and in-field support. The importance of node and network optimization, remote monitoring and troubleshooting is also different. The network in the parking deployment has a more complex structure but the cost of service is lower, while the network for the air quality application is simpler, but the reliability of the nodes and the network is more important because they are more costly to service during exploitation.

The toolset integrates in a unified framework several of the existing state of the art tools together with new productivity-enhancing tools developed during the project. The resulting framework supports well typical WSN development flows and is accessible to most application domain experts. The toolset allows the developer to efficiently focus on the application-specific elements by automating much of the time-consuming details that are not directly relevant for the application. Moreover, the modular structure of the framework and of the main composing tools simplify its maintenance and extension with new tools and IPs, either proprietary or open, for its continuous evolution and customization for specific needs.

Last but not least, the proposed framework is built around the concept of complete, end-to-end WSN application project, which simplifies the passage of data between the tools that support the various design phases, iterative exploration of different design variants and their comparison, as well as comparison and optimization of field results with respect to design specification and simulations.
